# Improving mental health by training the suppression of unwanted thoughts

**DOI:** 10.1126/sciadv.adh5292

**Published:** 2023-09-20

**Authors:** Zulkayda Mamat, Michael C. Anderson

**Affiliations:** ^1^ MRC Cognition and Brain Sciences Unit, University of Cambridge, Cambridge, UK.; ^2^ Behavioural and Clinical Neurosciences Institute, University of Cambridge, Cambridge, UK.

## Abstract

Anxiety, posttraumatic stress, and depression markedly increased worldwide during the COVID-19 pandemic. People with these conditions experience distressing intrusive thoughts, yet conventional therapies often urge them to avoid suppressing their thoughts because intrusions might rebound in intensity and frequency, worsening the disorders. In contrast, we hypothesized that training thought suppression would improve mental health. One hundred and twenty adults from 16 countries underwent 3 days of online training to suppress either fearful or neutral thoughts. No paradoxical increases in fears occurred. Instead, suppression reduced memory for suppressed fears and rendered them less vivid and anxiety provoking. After training, participants reported less anxiety, negative affect, and depression with the latter benefit persisting at 3 months. Participants high in trait anxiety and pandemic-related posttraumatic stress gained the largest and most durable mental health benefits. These findings challenge century-old wisdom that suppressing thoughts is maladaptive, offering an accessible approach to improving mental health.

## Teaser

Training people to suppress fearful thoughts improved mental health, especially for those with anxiety and post-traumatic stress.

## INTRODUCTION

Suppressing upsetting thoughts may promote mental health during adversity, and this capacity may be trainable. Historically, thought suppression has been considered maladaptive because of the century-old Freudian proposal that suppressed content persists in the unconscious mind, resurfacing indirectly through symptoms and dreams (*1*, *2*). According to modern theoretical accounts, even when thought suppression succeeds fleetingly, the suppressed content rebounds in accessibility and emotional intensity, amplifying a person’s distress (*3*, *4*). These clinical views remain unreconciled with neurobiological evidence that suppressing thoughts helps maintain mental health (*5*–*11*). For example, engaging the right lateral prefrontal cortex to suppress intrusive thoughts is associated with greater resilience to developing posttraumatic stress disorder (PTSD) after violent trauma (*11*), decreased anxiety about feared events (*12*), fewer distressing intrusions after viewing a traumatic film (*13*), and a tendency to forget suppressed content on both explicit (*14*, *15*) and implicit memory tests (*16*, *17*). Despite these potential benefits, direct causal evidence for how thought suppression affects mental health is lacking because the presumed risk of asking vulnerable populations to suppress distressing thoughts has discouraged experimental studies to determine its effects (*18*). If thought suppression improved mental health by reducing distressing thoughts and their emotional impact (*7*, *12*, *19*–*21*), then these discoveries could alter how we should treat anxiety, depression, and PTSD, a radical departure from current treatments that often strive to eliminate thought suppression (*22*–*26*).

We challenge the view that thought suppression worsens mental illness. We hypothesized, in contrast, that training people to suppress unpleasant thoughts in response to reminders would improve their mental health, even in people with anxiety, depression, and PTSD. People with these conditions suppress thoughts less effectively on laboratory measures, mirroring their intrusive symptoms in daily life (*11*, *27*–*31*). Such difficulties are often attributed to ineffective inhibitory control over memory and emotion, originating from structural, functional, or neurochemical deficiencies in the prefrontal cortex or the hippocampus (*32*–*40*). However, it remains unclear the extent to which thought suppression deficits also reflect modifiable factors, including inexperience with suppression, ineffective suppression strategies, or metacognitive beliefs about the impossibility of thought suppression that discourage its use, which are factors that may be remediated by repeated training that reveals suppression’s utility to the individual. We therefore causally tested how thought suppression affects mental health by training people to suppress their distressing thoughts about feared future events. We targeted participants’ fearful thoughts given the marked rise in anxiety, depression, and posttraumatic stress in the corona virus disease of 2019 (COVID-19) pandemic (*41*–*43*) and the potential benefit of ameliorating a key symptom of these conditions.

**Fig. 1.**
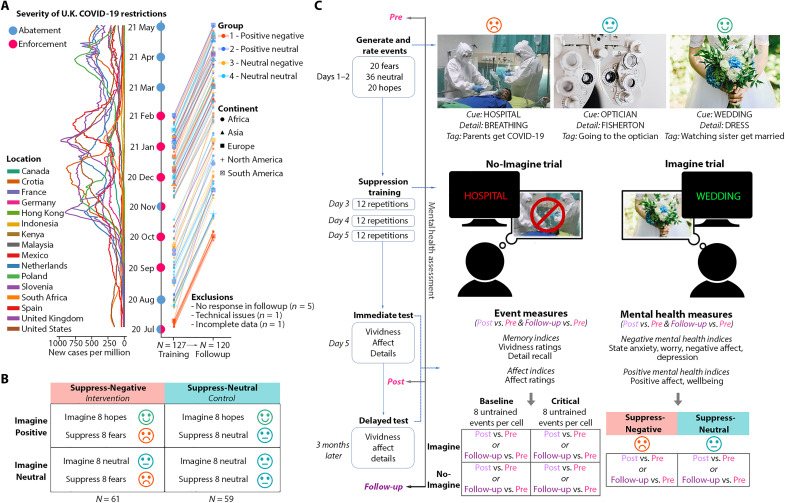
Experimental design and procedure. (**A**) One hundred and twenty participants from five continents participated in online suppression training and a follow-up assessment 3 months later during the COVID-19 pandemic. (**B**) We randomly assigned participants to suppress their fears (primary intervention; Suppress-Negative, *N* = 61) or neutral events (control; Suppress-Neutral, *N* = 59), with half of each group assigned to also imagine positive or neutral future events. (**C**) Participants first generated 20 fears, 36 neutral events, and 20 hopes (over 2 days) each with a cue, a key detail, a short tag line, and a brief description with more details (images in figure are only for illustration purposes and are all from free-to-use sources: The negative photo is by M. Majnun on Unsplash; the neutral photo is by K. Grabowska on Pexels, and the positive photo is by I. Lesyk on Pexels). They then rated event characteristics and had their mental health assessed; three days of retrieval suppression practice ensued, and each session was composed of 12 No-Imagine and 12 Imagine repetitions in response to No-Imagine and Imagine cues, respectively. No-Imagine cues (appearing in red) required participants to attend to the cue while suppressing retrieval of any imagery or thoughts; Imagine cues (appearing in green) required participants to imagine the event. Immediately after the final training session, we tested memory and affect for generated events and assessed mental health; we repeated these assessments after 3 months. Analyses of event measures focused on changes in memory and affect for each event after training compared to before (post versus pre or follow-up versus pre), as did measures of mental health. Event analyses permit assessment of the effects of imagination (Imagine row) or suppression (No-Imagine row).

Our study took place via individualized videoconferencing, anticipating a future with safe and accessible treatment delivery to participants worldwide. Before training, 120 adults from 16 countries listed feared future events of current concern to them, each with a cue word that reminded them of the event [Fig. 1; see table S1 (A to C) for participant and event characteristics]. They briefly described each fear and listed a single word denoting a central detail of what they typically imagine (see the “Procedure” section below). Via this procedure, participants also generated neutral and positive future events (i.e., “hopes”). During training, participants practiced the Imagine/No-Imagine (INI) task (*12*), which requires retrieval stopping (*5*, *14*, *15*), a particular form of thought suppression. Training took place over 3 days. On each trial of this task, participants confronted the cue to a future event for 4 s, and we asked them to imagine the event vividly (“Imagine” items) or to stop themselves from imagining it (“No-Imagine” items). Specifically, for No-Imagine trials, we asked participants to first recognize the feared event signified by the cue but, thereafter, to suppress retrieval of any thoughts or imagery about it. Participants practiced thought suppression extensively: Across 3 days, they suppressed every No-Imagine (or imagined every Imagine) event 36 times. After the final training session and following a 3-month delay, we tested how repeated thought suppression had affected the suppressed events. We evaluated risks of clinical concern by scrutinizing whether suppression promoted paradoxical increases [hereinafter termed ironic increases (*3*)] in memory or affect for participants’ fears or had instead enabled people to successfully forget these unwelcome thoughts.

Our central focus, however, was on whether training participants to suppress distressing thoughts causally affected their mental health. Worsening or improvement in mental health should be measurable as changes on clinical indices of depression, anxiety, worry, affect, and well-being (see the “Materials” section). We quantified such changes by measuring these clinical features before and after thought suppression training and after 3 months. To detect mental health effects unique to suppressing distressing thoughts, we compared changes in our indices for a group that suppressed feared events (the Suppress-Negative group, *N* = 61) to changes in a control group that suppressed neutral events (the Suppress-Neutral group, *N* = 59). Mental health changes in the Suppress-Neutral group provide a rigorous control against which to assess the unique effects of suppressing unpleasant content by quantifying general changes arising from other factors: from participants generating positive, negative, and neutral events at the study’s outset; from receiving thought suppression training in general; from placebo effects; or from socially interacting with an experimenter. If suppressing feared events harms or benefits participants, then mental health changes for the Suppress-Negative group should exceed those in the Suppress-Neutral group. In a complementary manipulation, we juxtaposed the mental health effects of suppressing fearful thoughts with those induced by positive imagery about hopeful future events. On Imagine trials, half of the participants imagined positive future events (Imagine-Positive), and half imagined neutral events (Imagine-Neutral), within each of the two Suppress groups (Fig. 1B). This manipulation enabled us to evaluate the mental health impact of thought suppression in relation to the effects of a popular approach to enhancing mood: positive thinking (*44*–*46*).

## RESULTS

### Impact of thought suppression on feared events

We first tested how thought suppression training affected memory for the suppressed events. We sought evidence for ironic increases in accessibility of suppressed content to quantify the risk of training people to suppress their fears. In healthy individuals, suppressing retrieval typically reduces later recall of the suppressed content, at least for simple words and images (*14*, *15*, *31*). This suppression-induced forgetting (SIF) effect is established by showing that the final recall of suppressed items is lower than that of Baseline items that were also encoded initially but that were neither suppressed nor retrieved after encoding. Consistent with this pattern, after suppression training, participants recalled the key detail of their personal events less often. Participants recalled fewer No-Imagine (i.e., suppressed) than Baseline items overall (*F*_1,118_ = 8.31, *P* = 0.005, η_p_^2^ = 0.066), and this effect did not vary across the Suppress-Negative and Suppress-Neutral groups (*F*_s_ < 1; table S2 and fig. S1). We also tested whether suppression altered events’ phenomenological quality by quantifying changes in reported vividness observed after training compared to before training. Suppressed events showed larger reductions in vividness (relative to pretraining) than did Baseline events (*F*_1,118_ = 6.61, *P* = 0.01, η_p_^2^ = 0.053), and this effect did not vary across the Suppress-Negative and Suppress-Neutral groups (*F* < 1; table S2 and fig. S1). Thus, as with laboratory stimuli, suppression reduced access to feared and neutral personal events, at least on a retention test immediately after suppression.

**Fig. 2.**
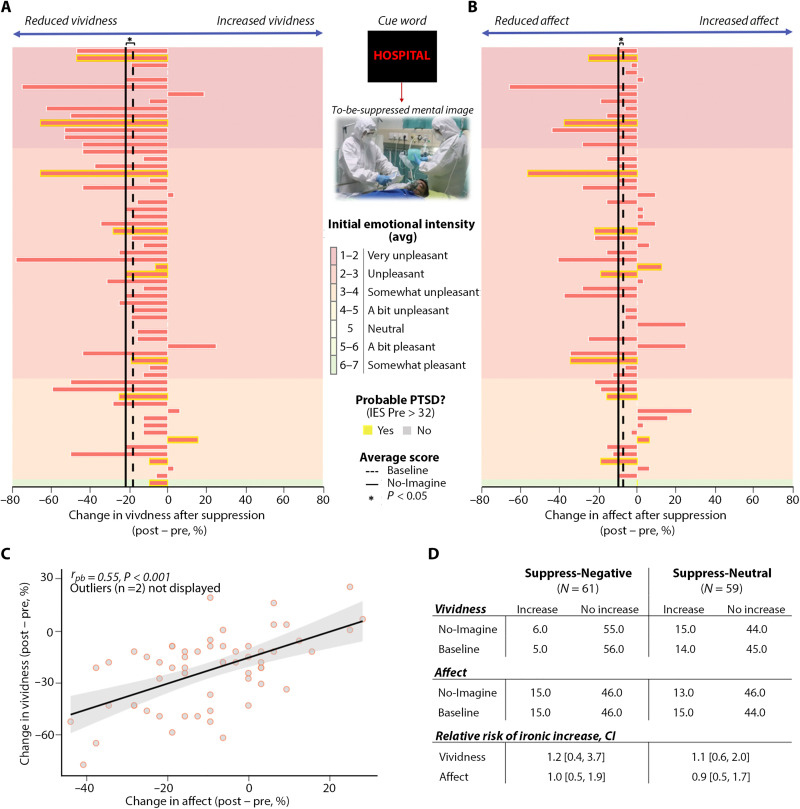
Changes in fear vividness and emotion after suppression reveal benefits, not ironic rebound. (**A**) Suppressing fears reduced their vividness on an immediate test for nearly all participants, irrespective of the initial emotional intensity of those fears or participants’ posttraumatic stress status; horizontal bars are individual participants’ average change in vividness [post–pre; percentage of maximum point (POMP) scores]. Participants are sorted vertically by the average emotional intensity of fears before training from least (bottom bars, light orange background) to most intense (top bars, darker red background); yellow highlights on bars indicate participants with probable PTSD. Across negative and neutral events, vividness reductions were significantly greater for No-Imagine than for Baseline items and did not vary reliably by event valence (mean changes indicated by solid black versus dotted vertical bars; see text for statistics) and are greater for participants whose events are initially more intense (*r* = 0.28, *P* = 0.027). (**B**) Suppressing feared events reduced their affective tone for most participants, irrespective of initial emotional intensity (as assessed on a separate nine-point scale; see the Supplementary Materials) and PTSD status; affect reductions were greater for No-Imagine than for Baseline items (solid black versus dotted vertical bars). (**C**) In the Suppress-Negative group, the more that suppression training reduced fear vividness, the greater the reduction in perceived fear intensity. (**D**) Suppressing future events rarely increased vividness or affect after training compared to before training, and the frequency of increases never exceeded those observed for Baseline events that were never suppressed, irrespective of event valence. The relative risk of an adverse event (a suppression-related increase in their fears’ vividness or affective intensity) was near to 1.0 (no increase in risk) for both the Suppress-Negative and Suppress-Neutral conditions [confidence intervals (in brackets) spanning 1.0 reflect nonsignificant changes in risk].

The preceding findings show that training people to suppress the retrieval of fearful thoughts does not lead those thoughts to rebound ironically on average. However, group-level memory declines for suppressed content could mask ironic effects for some individuals. Rebound effects could precipitate adverse events of clinical concern. To address the likelihood of rebound effects, we sought individual participants for whom the accessibility of suppressed details or fearful imagery increased on the posttraining measure compared to the pretraining measure. If ironic rebound effects are a serious concern, then such postsuppression increases should be common. Across the whole sample of 120 participants, No-Imagine items were associated with lower key detail recall (*F*_1,118_ = 109.4, *P* < 0.001, η_p_^2^ = 0.481) and reduced vividness (*F*_1,118_ = 103.608, *P* < 0.001, η_p_^2^ = 0.468) after, compared to before, training. Only 1 person of 120 showed higher detail recall for suppressed items after training. Of the 61 participants that suppressed fears, 6 reported increased vividness for No-Imagine items after training (Fig. 2A); of the 59 participants who suppressed neutral events, 15 reported increased vividness. Critically, however, these cases are unlikely to reflect ironic rebound effects: A similar number of participants reported increases for Baseline events (for Baseline items in the Suppress-Negative and Suppress-Neutral groups, 5 and 14 participants, respectively, showed increased vividness, and 1 and 4, respectively, showed increases in detail recall). We performed a relative risk analysis assessing the chance of increased accessibility after suppressing fears or neutral events, relative to their respective baselines (table S3A). Suppressing fearful or neutral thoughts did not increase the relative risk of ironic effects in either vividness or key detail recall (Fig. 2D and table S3A). Even when we examined individual feared events, the number of fears (per participant) showing increased vividness did not vary across the Baseline and No-Imagine conditions in any group (see table S3B).

We considered the possibility that ironic rebound effects only arise for emotionally intense fears of great concern to participants. To address this, we examined whether the degree to which suppression training reduced vividness or detail recall for No-Imagine fears varied according to participants’ initial emotional intensity ratings for those fears. Contrary to such concerns, greater fear intensity was associated with larger suppression-related reductions in vividness after training compared to before training (*r* = 0.283, *P* = 0.027; Fig. 2A). Initial emotional intensity was not associated to declines in detail recall (*r* = −0.068, *P* = 0.604).

The foregoing findings only address suppression’s impact immediately after training. Suppression might briefly benefit participants yet trigger hazardous hyperaccessibility of suppressed thoughts at longer delays. SIF was not detected after 3 months: At the follow-up, No-Imagine items were no longer recalled more poorly than were Baseline items, nor did they show greater declines in vividness compared to Baseline (*F* < 1 in all cases; table S4). One interpretation of this pattern is that suppression dissipated over the delay, causing an ironic rebound in fear accessibility. Contrary to this rebound effect, both memory for key details of No-Imagine items (*F*_1,118_ = 1731.8, *P* < 0.001, η_p_^2^ = 0.936) and the vividness of No-Imagine items (*F*_1,118_ = 27.74, *P* < 0.001, η_p_^2^ = 0.19) remained far lower, not higher, after 3 months than they were before suppression, with no indication of any special resurgence of these suppressed items. For example, after 3 months, in the Suppress-Negative group, numerically fewer participants showed increased vividness for No-Imagine events (follow-up − pre > 0, *N* = 14) than showed such increases for Baseline events (*N* = 21); the Suppress-Neutral condition showed no difference (*N* = 15 and *N* = 13 in the No-Imagine and Baseline conditions, respectively). As a result, for fears, the relative risk of ironic increases in vividness of suppressed events, relative to Baseline events suggested decreased, not increased risk, although this reduction was not significant (table S5A). Even when we examined individual events, the number of fears (per participant) that increased in accessibility did not differ between No-Imagine and Baseline conditions for any measure or group (*F*_s_ < 1; table S5). These findings suggest that suppression does not trigger rebound processes.

Last, we tested whether people reporting higher trait anxiety or pandemic-related posttraumatic stress suppressed event details or imagery less well. Participants reporting mental health symptoms may be especially vulnerable to rebound effects. We tested whether indices of mental health collected before training predicted declines in vividness and detail memory for No-Imagine fears in the Suppress-Negative group. Contrary to rebound concerns, suppression-related declines in vividness were greater, not smaller, the higher the reported trait anxiety (*r* = −0.264, *P* = 0.04) and the higher the reported depression (*r* = −0.324, *P* = 0.011) with the effect of depression surviving statistical correction (*47*). Vividness declines were not reliably related to pandemic-related posttraumatic stress (*r* = −0.11, *P* = 0.41; Fig. 2A). For memory of key details, neither higher trait anxiety (*r* = −0.116 *P* = 0.372) nor depression (*r* = −0.074, *P* = 0.57) predicted smaller declines in recall, and higher posttraumatic stress scores marginally predicted larger, not smaller, declines in recall (*r* = −0.25, *P* = 0.052). Similar though weaker relationships arose after 3 months (e.g., *r* = −0.21, *P* = 0.10, when relating pretraining trait anxiety to suppression-related declines in vividness), providing no evidence of increased vulnerability.

Together, these findings provide no indication that training people to suppress distressing thoughts of feared events triggered ironic rebounds in the accessibility of those thoughts on either an immediate or delayed test. Ironic effects were absent even for participants reporting high anxiety, depression, or posttraumatic stress, who instead often showed greater suppression-related declines.

### Reduced affective responses to feared events

Suppression training could intensify emotional responses to the suppressed content even if it fails to trigger ironic rebound effects in memory. If suppressed thoughts grow more distressing (*4*, *48*, *49*), adverse psychological events may occur. Theories of clinical disorders often posit escalating emotional responses to suppressed content, especially when thought suppression failures trigger negative self-appraisals (*26*, *48*, *49*). On the other hand, suppressing the retrieval of aversive pictures reduces later affective responses on both subjective and psychophysiological measures of emotion (*7*, *12*, *19*–*21*). To evaluate suppression training’s effect on subjective emotion, we tested whether participants’ ratings of their affective responses to suppressed events changed after suppression training compared to before training (post − pre). If suppression triggers ironic rebounds, affect ratings should grow more negative for suppressed events after training. However, affect ratings for No-Imagine events were lower after compared to before suppression (*F*_1,118_ = 37.018, *P* < 0.001, η_p_^2^ = 0.239; Fig. 2B). As with laboratory findings (*19*–*21*), declines were greater for No-Imagine than for Baseline events (*F*_1,118_ = 4.136, *P* = 0.044, η_p_^2^ = 0.034), and this affective SIF did not vary across Suppression groups (*F* < 1; table S6). Consistent with our memory indices, suppression-specific reductions in affective responses were not detected at 3 months, with no differences observed for No-Imagine and Baseline items (*F* < 1; table S6). However, at 3 months, affective ratings of No-Imagine events remained lower compared to pretraining, (*F*_1,118_ = 32.89, *P* < 0.001, η_p_^2^ = 0.218), establishing that time’s passage did not ironically increase subjective emotions to suppressed future fears. For feared events, suppression-related reductions in affect and vividness were robustly associated, suggesting that rendering thoughts less accessible weakens their capacity to instill anxiety (Fig. 2C).

In the Suppress-Negative group, few participants (*N* = 15) reported more anxiety about suppressed fears after training, and the number was the same for nonsuppressed baseline fears (*N* = 15). Given this pattern, the relative risk of a rebound in anxiety about suppressed fears was 1.0, indicating that the risk was no different from when no suppression had occurred (Fig. 2D and table S7). A similar pattern arose after suppressing neutral events (Fig. 2D and table S7). After 3 months, similar results arose for both groups, with no increased risk of ironic effects (table S7). Higher posttraumatic stress did not reliably predict smaller suppression-related affect declines (posttraumatic stress, *r* = −0.07, *P* = 0.62), and both higher trait anxiety (*r* = −0.232, *P* = 0.07) and depression (*r* = −0.32, *P* = 0.01) before training showed trends toward steeper declines in affect for suppressed fears, not smaller declines. Pretraining anxiety and depression were not reliably related to the decline in affect that fears showed after 3 months (*r* = −0.002 and *r* = −0.019, respectively). Last, the average emotional intensity of a participant’s fear at the study’s outset (as indexed by a distinct measure from that used to assess affect reductions; see the Supplementary Materials) did not predict the suppression-related affect reduction measured on immediate or delayed tests (immediate: *r* = 0.007, *P* = 0.94; delayed: *r* = 0.171, *P* = 0.062). Together, these findings show that suppression training caused no measurable rebound effects on emotional responses to feared events regardless of their initial intensity and often reduced affective responses on immediate tests. Suppression-related reductions in perceived fear were more prominent for people reporting higher trait anxiety and depression.

### Immediate effects of suppression on mental health

Detrimental thought suppression effects could emerge on measures of mental health despite their absence on explicit memory tests. As Freud suggested, suppressing distressing content may preserve it in the unconscious, where it could shape participants’ moods, perceptions, and actions (*1*, *2*). If suppressed content persists perniciously, then mental health indices should reveal the symptoms that it creates. To test this possibility, we calculated changes in reported depression, state anxiety, worry, positive and negative affect, and well-being from pretraining to posttraining. We did this separately for the Suppress-Neutral and Suppress-Negative groups to isolate unique effects of suppressing fears, beyond those of training suppression more generally.

**Fig. 3.**
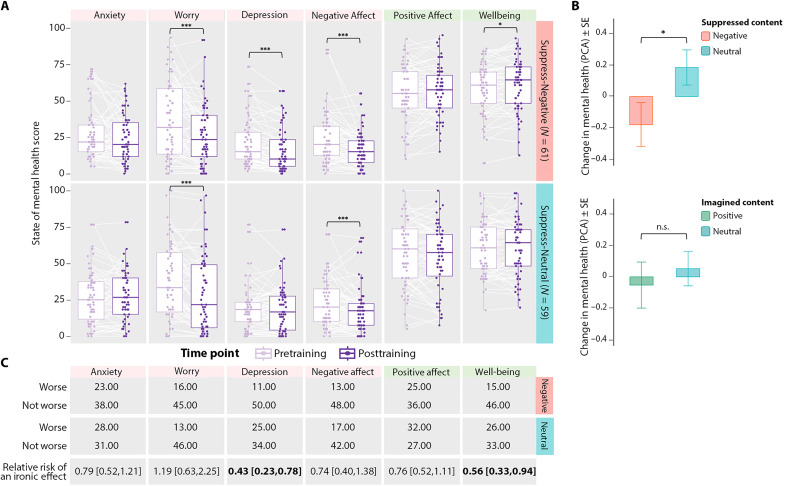
Suppressing fears both improves and protects mental health. (**A**) Training participants to suppress fears (upper half, red label) significantly reduced worry, depression, and negative affect and increased well-being after training (right-hand bar within each panel) compared to before training (left-hand bar within each panel); training at suppressing neutral events (lower half, blue label) reduced worry and negative affect. Individual participants are indicated by dots connected by white lines; boxes reflect interquartile range, and lines reflect median scores. (**B**) A PCA on change scores (post − pre) for our four negative and two positive measures yielded a latent variable that revealed more mental health improvement (more negative scores) in the Suppress-Negative than the Suppress-Neutral group; an analysis contrasting participants who imagined positive future events versus neutral events revealed no reliable difference in mental health changes on this latent variable. (**C**) Suppressing fears generally reduced the chances of an adverse event (a worsening of mental health; i.e., post > pre for negative indices; post < pre for positive indices) compared to suppressing neutral events on nearly every mental health index, significantly so for depression and well-being, which exhibited 57% and 44% decreases in the chances of an adverse event, respectively [confidence intervals (in brackets) spanning 1.0 reflect no change in relative risk; significant reductions are indicated in boldface]. ns, not significant. *P < 0.05; ***P < 0.001.

Mental health improved on most measures after training people to suppress distressing thoughts (Fig. 3A). In contrast, training people to suppress neutral thoughts did not improve most measures, except worry and negative affect, which showed comparable gains in both groups (Fig. 3A). To compare mental health benefits across training groups, we conducted a principal components analysis (PCA) on change scores (post − pre) for all six measures to derive a latent construct reflecting broad mental health changes. Using this index, participants trained to suppress fears experienced more improvement in mental health than did those trained to suppress neutral events (*t*_118_ = −2.012, *P* = 0.045; Fig. 3B). Greater improvement for the Suppress-Negative group cannot be attributed to preexisting group differences on mental health measures; the nature of the positive, neutral, or negative events generated; or to the amount of SIF they exhibited [see tables S1 (B and C) and S2]. This effect suggests that training the suppression of distressing events benefitted participants more than did training suppression itself. Supporting this interpretation, the extent to which training reduced affect for suppressed fears predicted more negative scores (better outcomes) on our mental health latent variable (*r* = 0.27, *P* = 0.034).

To be sure that overall training benefits did not disguise ironic effects in the Suppress-Negative group, we quantified how often mental health declined after training for individual participants. In relative risk analyses, no mental health index showed significantly greater risk of ironic effects after suppressing fears than after suppressing neutral thoughts, with most measures indicating numerically less frequent adverse changes for the former (Fig. 3C and table S8). Notably, suppressing fears reduced the risk of an ironic decline in well-being by 44.2% [95% confidence interval (CI) = [0.33, 0.94]] and of a worsening of depression by 57.4% (95% CI = [0.23, 0.78]), relative to suppressing neutral events (Fig. 3C). This finding demonstrates that suppressing unpleasant thoughts plays an important role in protecting people from declining mental health (not simply improving it), aiding in resilience to adverse conditions.

Unlike suppressing fears, training participants to imagine positive and joyful future events conferred little unique benefit. We compared mental health changes after training participants to imagine hopeful future events (*N* = 60) to those after training participants to imagine neutral events (*N* = 60). Because half of each group suppressed negative and half suppressed neutral events, positive imagery’s impact can be examined with suppression valence held constant. Although practice at imagining positive events reduced worry and negative affect after training, comparable benefits arose when people practiced imagining neutral events, suggesting that improvements did not stem from positive imagery. Our PCA latent variable for mental health change yielded no reliable advantage for the Imagine-Positive over the Imagine-Neutral group (*t*_118_ = −0.57, *P* = 0.569; Fig. 3B). Moreover, increased positive affect for Imagined scenarios was not related, across participants, to mental health improvement (as indexed by the PCA component) for either imagination group (*r* = −0.04 and −0.13 in the Imagine-Positive and Imagine-Neutral groups, respectively). The lack of specific benefits of positive imagination cannot be attributed to an ineffective manipulation: Imagined events showed robust increases in detail recall (*F*_1,118_ = 34.20, *P* < 0.001, η_p_^2^ = 0.225; interaction with valence, *F* < 1), vividness (*F*_1,118_ = 197.26, *P* < 0.001, η_p_^2^ = 0.626; interaction with valence, *F* < 1), and subjective ratings of affect (*F*_1,118_ = 43.62, *P* < 0.001, η_p_^2^ = 0.27; interaction with valence, *F*_1,118_ = 7.73, *P* = 0.006, η_p_^2^ = 0.062; see figs. S1 and S2). These increases in vividness, memory, and affect for specific positive future events, however, did not uniquely improve participants’ mental health beyond imagining neutral events.

### Suppression training effects in anxious and traumatized participants

Despite the mental health benefits in our full sample, suppression training may harm those with anxiety, depression, and posttraumatic stress. Pathological worry, rumination, and intrusive memories in these conditions are often attributed to neurobiological deficiencies that may be difficult to rectify with training (*11*, *27*, *35*–*40*). People with these conditions may suffer ironic rebound effects. To address this possibility, we tested whether pretraining trait anxiety and pandemic-related posttraumatic stress predicted mental health improvement (posttraining–pretraining).

**Fig. 4.**
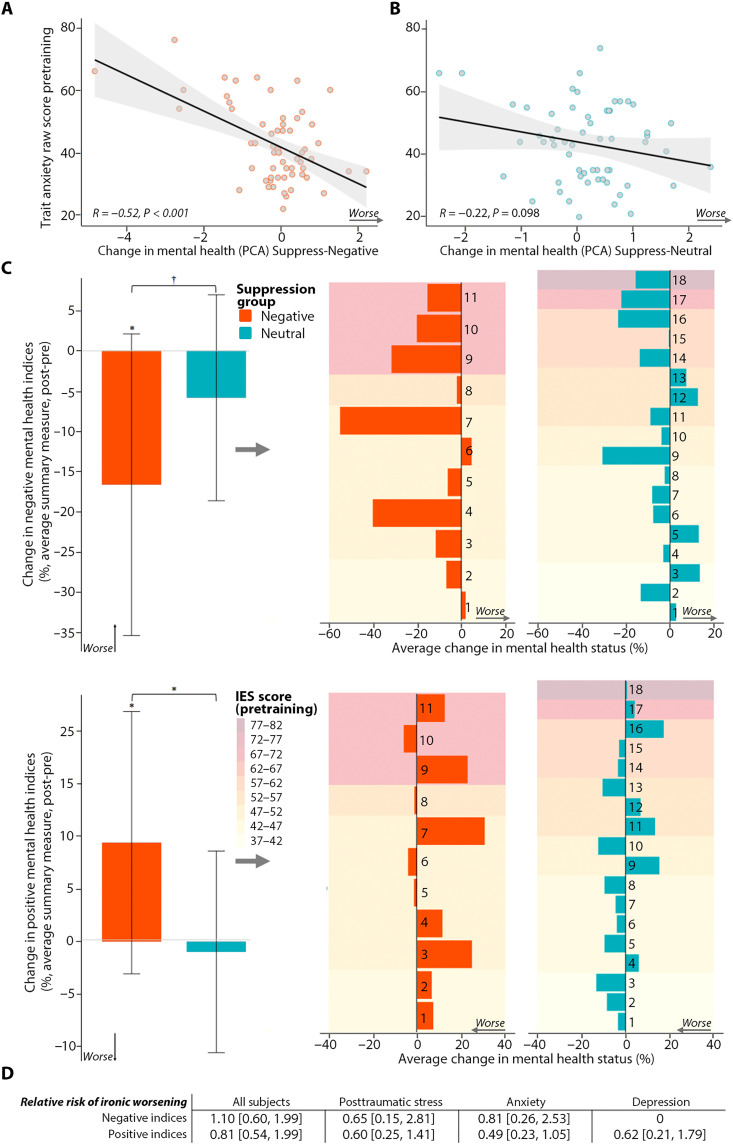
Mental health benefits of suppression training in symptomatic participants. (**A**) Higher pretraining trait anxiety scores [Trait portion of the State-Trait Anxiety Inventory Form Y-1 (STAI-Trait)] predicted greater mental health benefits for participants in the Suppress-Negative group on our PCA-derived mental health change latent variable. (**B**) Trait anxiety did not predict mental health changes in the Suppress-Neutral group. (**C**) Participants with likely pandemic-related PTSD [Impact of Events Scale–Revised (IES); IES score > 32] showed mental health improvement on both negative mental health indices (state anxiety, depression, worry, negative affect) and positive indices (well-being and positive affect) after training to suppress fears (Suppress-Negative group) compared to participants trained to suppress neutral events (Suppress-Neutral group). Left: POMP scores averaged over the negative scales (top) and positive scales (bottom). Right: Individual participants with likely PTSD in the Suppress-Negative group (left, red) and the Suppress-Neutral group (right, blue), sorted by IES score (least to greatest, from bottom to top) to illustrate that benefits occurred irrespective of PTSD symptom severity. (**D**) The relative risk of adverse events (a worsening of mental health after training) was never greater after suppressing fears compared to suppressing neutral events whether considering all subjects or subgroups with likely PTSD (IES, >32), substantial anxiety (STAI-Trait, >45), or depression [Beck’s Depression Inventory II (BDI), >19] [confidence intervals (in brackets) that include 1 indicate no increased risk]. *P < 0.05 and †*P* ~ 0.05.

Contrary to the foregoing concerns, participants reporting higher trait anxiety and posttraumatic stress benefitted the most from suppressing their distressing thoughts. After correcting for multiple comparisons, higher trait anxiety predicted larger reductions in worry, negative affect, and depression and larger increases in positive affect and well-being [*r* = −0.48, −0.61, −0.30, 0.32, and 0.3, respectively, significant after Benjamini-Hochberg correction (*47*); see fig. S3A]. Trait anxiety also predicted larger reductions in state anxiety (*r* = −0.26, *P* = 0.04), although this did not survive statistical correction. Participants who reported greater pandemic-related posttraumatic stress showed similar though weaker relationships for most mental health indices, and only the benefits to well-being (*r* = 0.34, *P* = 0.007) survived statistical correction (fig. S3B). Higher trait anxiety (*r* = −0.52, *P* < 0.00001; Fig. 4A) and posttraumatic stress (*r* = −0.35, *P* = 0.0068) predicted greater improvement (more negative scores) on our PCA-derived mental health change latent variable. These correlations did not occur in the Suppress-Neutral group: Trait anxiety or posttraumatic stress did not correlate with most individual measures (fig. S3, A and B) or with our mental health change latent variable [*r* = −0.22, *P* = 0.1 and *r* = −0.22, *P* = 0.1 for trait anxiety (Fig. 4B) and posttraumatic stress, respectively]. The correlation between trait anxiety and mental health improvement (Fig. 4A) was greater for those suppressing negative than neutral content (one-tailed, Fisher’s *z* = 1.894, *P* = 0.029), showing that the former correlation is not the inevitable result of participants with the highest scores regressing to the mean, as the opportunity for this was equivalent in both groups. Thus, people suffering from higher trait anxiety or posttraumatic stress benefitted more from suppression training but only if trained to suppress distressing content.

Despite the foregoing associations, truly severe anxiety or posttraumatic stress might put participants at elevated risk of ironic rebound effects. To test this possibility, we isolated 23 participants in our Suppress-Negative group with trait anxiety scores warranting clinical concern (STAI-Trait, >44) (*50*) and 11 participants with Impact of Event Scale scores that reflect likely PTSD (IES > 32). On every mental health measure except state anxiety, participants in the Suppress-Negative group with high trait anxiety showed significant benefits (see table S9A) with the index for worry dropping, on average, 17 points (a 44% reduction). Participants with high posttraumatic stress scores showed similar benefits (table S9B; see Fig. 4C for an analysis based on summary measures). Similar subgroups in the Suppress-Neutral condition showed few of these benefits (table S9, A and B, and Fig. 4C), with depression, well-being, and positive affect showing the most reliable differences across the Suppress-Negative and Suppress-Neutral groups (table S9, A and B). These findings suggest that benefits arose from suppressing negative content. A relative risk analysis indicated that participants high in trait anxiety or with likely PTSD had numerically lower risk of ironic reversals on most mental health indices after suppressing negative content compared to neutral content (see Fig. 4D for an analysis based on summary measures), with significantly reduced risks for reversals in well-being and positive affect (table S10, A and B). Training the suppression of worries thus protected participants with likely PTSD or anxiety disorders from declines in well-being or positive affect, in notable contrast to ironic rebound predictions.

### Sustained benefits of suppression after 3 months

Improved mental health after training may be transient. SIF of suppressed fears on indices of memory, vividness, and affect were not detected at the 3-month delay. However, suppressed fears may remain less intrusive after 3 months, even if they are voluntarily recallable. To examine the durability of mental health benefits, we first examined changes on each of the six individual indices 3 months after training.

After 3 months, participants trained to suppress their fears continued to show reduced depression (*F*_1,60_ = 11.3, *P* < 0.001, η_p_^2^ = 0.158) and a trend toward reduced negative affect (*F*_1,60_ = 2.982, *P* = 0.089, η_p_^2^ = 0.047), relative to pretraining levels (fig. S4). Those trained to suppress neutral events showed neither of these effects (depression: *F*_1,58_ = 1.473, *P* = 0.23, η_p_^2^ = 0.025; negative affect: *F*_1,58_ = 2.042, *P* = 0.158, η_p_^2^ = 0.034); they did, however, show reduced worry, as they had immediately after training (*F*_1,58_ = 9.059, *P* = 0.004, η_p_^2^ = 0.135). Neither group showed reliable benefits on state anxiety, positive affect, or well-being (*P* > 0.25 in all cases). A PCA on change scores (follow-up − pre-training) for our six mental health indices revealed a latent variable like the one derived on the basis of our immediate assessment. The Suppress-Negative and Suppress-Neutral groups did not differ on this global measure (*F* < 1), suggesting that training people to suppress distressing thoughts provided no sustained aggregate mental health advantage over training them to suppress neutral thoughts, when considering all participants (fig. S4).

**Fig. 5.**
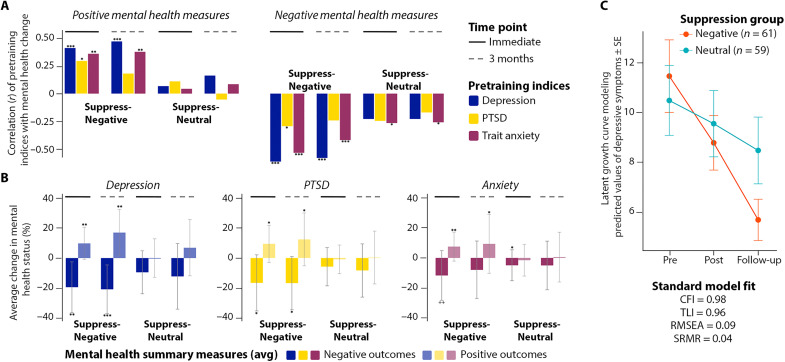
Depression, anxiety, or posttraumatic stress symptoms before training predict mental health benefits of fear suppression immediately after training and after 3 months. (**A**) Greater pretraining trait anxiety (maroon bars), pandemic-related posttraumatic stress (yellow bars), and depressive symptoms (dark blue bars) predicted larger improvements in positive mental health indices (left half of the panel) and negative mental health indices (right half of the panel) on our immediate assessment after training (solid line above bars) and 3 months later (dashed line above bars) but only when people were trained to suppress fears (Suppress-Negative) and not neutral events (Suppress-Neutral). Correlations (*r* values) are plotted on the *y* axis; significance is indicated by asterisks (*P < 0.05, **P < 0.01, and ***P < 0.001). Positive mental health is a composite of well-being and positive affect (averaged POMP scores); Negative mental health is a composite of the average POMP scores of the negative indices. (**B**) Subgroups with higher depression symptoms, likely PTSD, or anxiety showed significant improvements on negative mental health indices (darker shades, extending downward) and positive indices (lighter shades, extending upward) during the immediate test (post − pre; bars below solid lines) and the 3-month follow-up (follow-up − pre; bars below dotted lines). Improvements occurred only after suppressing fears (Suppress-Negative), not after suppressing neutral events (Suppress-Neutral). (**C**) A latent growth curve modeling (LGCM) analysis across our three time points (Pre, Post, and Follow-up) revealed greater improvement in depression after suppressing fears than after suppressing neutral events during training (*y* axis values are predicated values of depressive symptoms based on the LGCM model).

The foregoing analysis of the entire sample includes many participants with good initial mental health and may obscure durable improvement in symptomatic participants, who had shown the largest gains after training. To address this possibility, we correlated trait anxiety and posttraumatic stress indices before training with mental health improvement after 3 months. After correcting for multiple comparisons, higher trait anxiety before training (indicated by STAI-Trait scores) was associated with larger reductions in worry, negative affect, depression, and state anxiety and larger increases in positive affect and well-being [*r* = −0.33, −0.41, −0.46, −0.31, 0.37, and 0.33, respectively, significant after Benjamini-Hochberg correction (*47*); see fig. S3A and Fig. 5A for analyses based on summary measures]. Pandemic-related posttraumatic stress before training (IES) showed similar associations, although only depression remained significant after statistical correction (fig. S3B and Fig. 5A). Both higher trait anxiety (*r* = −0.49, *P* < 0.0001) and posttraumatic stress (*r* = −0.28, *P* = 0.028) predicted greater improvement on our PCA-derived mental health change latent variable at 3 months. In contrast, in the Suppress-Neutral group, correlations were not significant for either individual indices (fig. S3, A and B, and Fig. 5A) or our PCA-derived latent variable (*r* = 0.05 and *r* = 0.08 for trait anxiety and posttraumatic stress, respectively). These correlations were again significantly lower than in the Suppress-Negative condition (one-tailed, Fisher’s *z* = −3.13, *P* < 0.001 for anxiety and *z* = 1.96, *P* = 0.025 for PTSD), showing that the latter correlations are not the inevitable result of participants with the highest scores regressing to the mean, as the opportunity for this was equivalent in the Suppress-Neutral group.

The foregoing findings suggest that people with likely PTSD or high trait anxiety continue to reap benefits from suppression training after 3 months, but only if they suppressed distressing content. In the Suppress-Negative group, the overall mental health benefit gained after 3 months (PCA-derived latent variable) was predicted by how much suppression had reduced negative valence for fears immediately after training completed (*r* = 0.34, *P* < 0.006). Thus, successfully suppressing affect for fears was linked to enduring benefits. Participants qualifying for a provisional PTSD diagnosis (IES score > 32, *N* = 11) showed significant mental health benefits 3 months after suppressing fears: After correcting for multiple comparisons (Benjamini-Hochberg correction), they showed improved depression (*F*_1,10_ = 15.306, *P* = 0.003, η_p_^2^ = 0.605), negative affect (*F*_1,10_ = 7.822, *P* = 0.019, η_p_^2^ = 0.439), anxiety (*F*_1,10_ = 7.443, *P* = 0.021, η_p_^2^ = 0.427), and well-being (*F*_1,10_ = 7.379, *P* = 0.022, η_p_^2^ = 0.425) (see table S11A and Fig. 5B for analyses based on summary measures). No significant improvements arose for a similarly composed group (*N* = 18) who suppressed neutral scenarios (see table S11A and Fig. 5B), with the amount of mental health improvement on our latent variable greater after suppressing fears than neutral events (*P* < 0.05). Participants reporting high anxiety (STAI-Trait, >44, *N* = 23) also showed improved depression (*F*_1,22_ = 8.051, *P* = 0.01, η_p_^2^ = 0.268) and positive affect (*F*_1,21_ = 6.41, *P* = 0.019, η_p_^2^ = 0.226) and similar trends on other measures if they suppressed fears (see table S11B and Fig. 5B). Neither group of vulnerable participants showed a significant increase in ironic reversals after suppressing fears compared to suppressing neutral events, and in most cases, this risk was reduced (table S10, A and B).

Estimating suppression training’s long-term impact on mental health is challenging because adverse events can arise during the 3-month delay, especially given the global pandemic’s multiple waves. Because we ran the Suppress-Negative and Suppress-Neutral groups concurrently, variability introduced by such events should be similar across groups. Nevertheless, estimates of how training effects changed over time should consider this variable impact across participants and time points. To characterize how training affected mental health over time, taking this variability into account, we conducted latent growth curve analyses on individual measures. In these analyses, we included estimates of the pandemic’s recent impact on participants both before training and at our 3-month follow-up via IES scores. Given the number of parameters to be estimated by each model, only the model for our depression index converged. This model revealed that, over time, depression declined over the three time points for both the Suppress-Negative and Suppress-Neutral groups but that it did so more rapidly for the Suppress-Negative group (one-sample *t* test of the predicted values of the latent variable slope, based on group, *t* = −6.1608, df = 107, *P* = 1.296 × 10^−8^; Fig. 5C). These findings suggest that training people to suppress fearful thoughts benefitted depression over time more than did training them to suppress neutral thoughts. More broadly, our delayed assessment suggests durable benefits of suppression training on multiple indices for symptomatic participants, contrary to concerns over ironic rebound effects.

### Perceived effects of suppression

Participants’ subjective reactions could determine whether they adopt thought suppression skills into their lives. We asked about participants’ experiences with suppression, during and after training, focusing on those trained to suppress fearful content.

During training, participants reported improved ability to control fearful thoughts in response to reminder cues. They reported slight to moderate success during the first training session (*M* = 2.7 on a five-point scale); by the end of training on day 3, they reported being extremely effective (*M* = 4.5), a significant improvement (*F*_1,50_ = 221.7, *P* < 0.001, η_p_^2^ = 0.816), with 57% of the participants selecting the maximum rating. No participant showed an ironic decline. Seventy-three percent of participants rated themselves as surprised or very surprised by this ability, and on a free report question about major insights derived from training, 67% of comments remarked that the benefits of suppressing their thoughts was the biggest discovery, with only 10% of participants commenting on the value of positive imagination during Imagine trials.

**Fig. 6.**
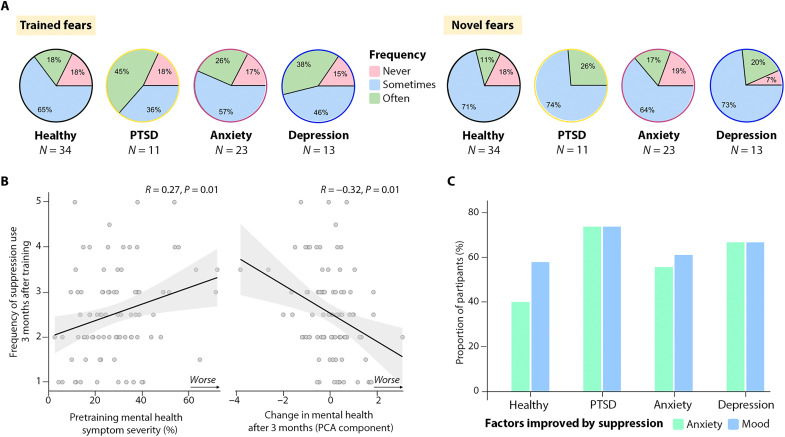
Participants spontaneously suppressed fears over the 3-month delay, concluding that it improved their mood and anxiety. (**A**) Most participants in the Suppress-Negative group reported using suppression over the delay both for trained (left side) and novel fears (right side), irrespective of whether pretraining indices suggested good mental health or pandemic-related PTSD, anxiety, or depression. Values reflect the percentage of people claiming to use suppression either Never (pink), Sometimes (light blue), or Often (green). (**B**) Pretraining mental health symptom severity (*x* axis, composite based on the average of trait anxiety, BDI, and IES) predicted the frequency with which participants reported using suppression during the delay (left); suppression use predicted increases in mental health benefits (right). (**C**) Healthy and symptomatic participants reported improved mood and anxiety that they directly attributed to suppression, with the largest improvements in highly symptomatic participants.

These perceptions translated into participants’ use of thought suppression during the 3-month delay, despite receiving no instructions to do so. After 3 months, 82% of all participants reported having used thought suppression for the trained fears and 80% for novel fears, a pattern that was true for both healthy and symptomatic participants (Fig. 6A), including those with probable PTSD due to the pandemic (81% for trained fears; 100% for novel thoughts). Increased symptom severity before training (reflecting a composite of trait anxiety, posttraumatic stress symptoms, and depression symptoms) predicted spontaneous use of suppression over the 3-month delay (*r* = 0.27, *P* = 0.01; Fig. 6B), and suppression use robustly predicted how much mental health had improved after this delay on our PCA-derived mental health measure (*r* = −0.32, *P* < 0.001; Fig. 6B). At the follow-up, 87% of all participants reported finding suppression useful. Among participants with probable PTSD, 82% reported reduced anxiety and 63% reported improved mood that they attributed specifically to learning thought suppression (Fig. 6C), with 27% reporting that they had “much better mood” and were “much less anxious” because of the training. No participants with likely PTSD or with clinically concerning anxiety reported ironic worsening of mood or anxiety during the delay period. These findings are the opposite to what should arise according to conventional wisdom about paradoxical suppression effects and indicate high participant endorsement for the benefits of suppressing distressing thoughts (see table S12 for verbal reports from all 61 participants in the Suppress-Negative condition about their experiences with suppressing fears over the 3-month delay).

## DISCUSSION

The mnemonic and affective patterns that we observed after training participants to suppress distressing thoughts support a role of thought suppression in protecting and enhancing mental health, in which suppression reduces (i) immediate awareness of distressing content, (ii) later voluntary access to details and imagery of feared scenarios, and (iii) the subjective distress associated with feared scenarios. When successful, these regulatory impacts prevent suppressed content from driving worrying, rumination, and other forms of repetitive thinking that amplify anxiety, depression, and posttraumatic stress. Such reductions, coupled with increased perception of control over their thoughts (table S12), improved people’s well-being. Participants voluntarily applied suppression to their targeted fears and to new fears in the 3 months after training, yielding enduring mental health gains for symptomatic participants.

The current findings challenge the pervasive view that thought suppression plays a key role in the pathogenesis of mental health disorders. We observed no evidence that training people to suppress distressing thoughts increased the risk of paradoxical rebound effects on any of our mnemonic, affective, or mental health indices; risk did not increase, regardless of delay; the affective intensity of feared events; or participants’ level of trait anxiety, depression, or posttraumatic stress at the outset of training, contrary to concerns. The negative assessment of thought suppression among clinical psychologists originated in the historical Freudian view that suppressed contents persist in influencing us unconsciously (*1*, *2*) and has been continued by theoretical claims about the ironic effects of thought suppression (*3*, *4*). Clinical concern about ironic effects has been motivated by data from the White Bear thought suppression task, which, unlike retrieval suppression, requires participants to remember and monitor for a specific forbidden thought (White Bears) as they strive to suppress that very thought. Meta-analyses of this task suggest a small rebound effect immediately after thought suppression ends (*51*, *52*), an effect that does not survive correction for small study bias (*51*). Our data indicate that retrieval suppression, which instead seeks to interrupt the progression from cues to unwelcome thoughts, does not carry this rebound risk, and we suggest that it better captures thought suppression as it occurs naturally (*8*, *53*). We hypothesize that retrieval suppression succeeds by recruiting inhibitory control mechanisms (*14*, *15*, *54*, *55*) in tandem with the circuitry underlying fear extinction, to inhibit mnemonic and affective responses in parallel (*7*, *8*, *12*, *53*, *56*). By training people to persistently confront reminders that reactivate their fearful thoughts [a key precursor to memory disruption (*57*)] and then driving them to suppress awareness of the associated memory, our protocol combines active forgetting of distressing imagery (*12*, *58*–*60*), with the controlled recruitment of extinction circuitry, believed critical in adjusting emotional responses to threat (*56*, *61*–*66*). In a classical fear extinction paradigm involving electrical shocks, asking people to suppress retrieval of the fearful shock during extinction learning benefits the durability and generalization of extinction (*66*).

Whatever mechanisms underlie the current benefits, our experiment shows that suppression training can improve mental health for those suffering from symptoms of anxiety and PTSD. In doing so, we provide an alternative lens on the origins of persistent intrusive thinking in these disorders. If participants high in trait anxiety or posttraumatic distress had suffered from structural or neurochemical deficits in the prefrontal cortex or other brain structures needed for thought suppression, it is unlikely that a 3-day training regimen would have improved control over their symptoms to the degree that it did. The training’s clearest impact was to increase participants’ awareness of thought suppression and to vividly illustrate its value in regulating their most distressing fears. Thus, our training may have eliminated a metacognitive gap or instead may have altered false beliefs about the dangers of thought suppression that limited its use, revealing the latent ability that our participants had but did not use. Many participants reported surprise at their ability to suppress and how much it improved their mood. The ability of our short suppression training intervention to substantially improve mental health in those with high anxiety or posttraumatic stress raises doubts about the emphasis on anatomical deficiencies in these conditions and suggests that the fraction of psychiatric disorders arising from behaviorally treatable causes is higher than clinical neuroscientists typically assume. Deficient prefrontal cortex activation in brain imaging studies of psychiatric disorders can be misinterpreted as a neurobiological deficiency when it may often simply indicate a treatable failure to engage intact abilities. Identifying patients that strongly benefit from suppression training could better isolate nonresponsive individuals with true neurobiological deficits, improving scientific study of these conditions and enabling targeted interventions tailored to individual needs. More broadly, the substantial and durable mental health benefits, safety, high endorsement, spontaneous use, and accessible delivery make suppression training a promising and scalable intervention on its own or as a neurobiologically grounded complement to standard treatments such as exposure or cognitive behavioral therapy.

## MATERIALS AND METHODS

### Experimental design

To determine how suppressing distressing content affected mental health, we manipulated the negativity of the content that participants suppressed during No-Imagine trials on suppression training days. We randomly assigned participants to suppress one of two types of content: Participants suppressed either their fears (primary intervention; Suppress-Negative, *N* = 61) or neutral events (control; Suppress-Neutral, *N* = 59). As a secondary variable, we manipulated the positivity of the events that participants imagined during Imagine trails on training days. We randomly assigned half of the participants to imagine positive (Imagine-Positive, *N* = 60) or neutral (Imagine-Neutral, *N* = 60) future events. Thus, our intervention constituted a 2 × 2 between-participants design with negativity of suppression content as the primary manipulation.

To implement this design, participants first generated 20 fears, 36 neutral events, and 20 hopes (over 2 days) each with a cue, a key detail, a short tag line, and a brief description with more details (for a more complete description of the methods than is provided by this broad summary, see the “Materials” and “Procedures” sections below). They then rated event characteristics and had their mental health assessed; three days of retrieval suppression practice ensued, each day composed of 12 No-Imagine and 12 Imagine repetitions in response to No-Imagine and Imagine cues, respectively. No-Imagine cues (appearing in red) required participants to attend to the cue while suppressing retrieval of any imagery or thoughts; Imagine cues (appearing in green) required participants to imagine the event (see Fig. 1 for illustrations). Immediately after the final training session (and again after 3 months), we tested memory and affect for generated events and assessed mental health.

To determine how suppression or imagination affected the events themselves (rather than mental health), we compared indices sensitive to event memory and affect across experimental conditions with their respective control events. Control events were other events generated by the participant that had the same valence as the suppressed content and that were matched algorithmically on vividness, intensity, and other event attributes (see the “Materials” section). To probe the SIF effect in event memory, we computed the percentage of key details correctly recalled for No-Imagine items and No-Imagine Baseline items. We judged SIF as having occurred if participants recalled fewer No-Imagine items than No-Imagine Baseline items. To determine whether imagination facilitated recall of imagined items, we compared key detail recall for Imagine items to Imagine-Baseline items. To probe the SIF effect in vividness, we computed change scores for participants’ vividness ratings (posttraining − pretraining), expressing these scores as percentages [percentage of maximum point (POMP) scores; see the “Statistical analyses” section] for No-Imagine and No-Imagine Baseline items. We judged SIF as having occurred if participants reported a larger change in vividness for No-Imagine than for No-Imagine Baseline items. We performed an analogous analysis to detect suppression-induced changes in affective intensity. We computed the impact of imagination on vividness and affective intensity in analogous fashion but using Imagine and Imagine-Baseline scores instead of No-Imagine and No-Imagine Baseline scores. We conducted similar analyses on the immediate assessment and after the 3-month delay. For delayed vividness and affect analyses, we computed change scores by comparing participants’ follow-up ratings after 3 months to their pretraining ratings (follow-up − pre).

### Participants

One hundred and twenty-seven people took part initially, of whom we excluded seven participants (five because of not being able to contact for follow-up, one because of issues connecting to Zoom, and one because of incomplete questionnaire data). The final sample included the 120 participants for whom we had complete data in the immediate and follow-up sessions (93 females, age: μ = 27.41, SD = 10.21; see table S1A for further demographic characteristics of participants).

In session 1, we randomly assigned participants to conditions, which differed in the valence of the events that participants needed to suppress during training (i.e., Suppress-Negative or the Suppress-Neutral), each of which further differed in events that participants needed to imagine during training (Positive or Neutral events). We assigned participants via blocked randomization, such that we ran each condition once for every four participants, ensuring that we matched the number of participants in groups as we ran the study. We determined the sample size for each of the four groups based on a priori power analysis with G*Power 3.1 software (*67*) [*f* = 0.27 (*68*), α = 0.05, 1 − β = 0.8, number of groups = 1, number of measurements = 2, correlation among representative measures = 0.49 (*12*)] using data from (*12*) as basis. This indicated a required sample of 30 participants per experimental group, resulting in 120 participants for the entire experiment. Primary analyses focused on comparisons between the Suppress-Negative and Suppress-Neutral groups.

We recruited participants from the MRC Cognition and Brain Sciences Unit (CBU) participant panels with the constraint that they had no prior participation in research involving the Think/No-Think (TNT) or the INI paradigms. We also recruited a small set of participants via online study advertisements on Facebook and Twitter and via word of mouth from previous participants. We paid participants according to the rates set for behavioral experiments at the MRC CBU (£6/hour). Exclusion criteria included a history of attention deficit disorder, reading disability, or lack of English fluency. Non-native English speakers could participate if they were fluent in English from early childhood and scored above 90% on the Cambridge General English Assessment (*69*). The Cambridge Psychology Research Ethics Committee approved the study, with all participants providing informed consent digitally.

### Remote testing

To allow international participation during the COVID-19 pandemic, we conducted the study online via the videoconferencing platform Zoom (*70*). Because Zoom meetings are accessible via a web browser, no software installation was required. For one participant, we used Skype instead because of technical difficulties. Videoconferencing allowed the experimenter to share their screen during training. To mimic laboratory studies, we required that participants to use a desktop or laptop for all sessions except session 1. Because this introductory session did not involve experimental tasks, we allowed participants to use an iPad or a smartphone if they wished. We required all participants to remove distractions from their environment and to keep their cameras on during all the sessions.

### Materials

Each participant generated 76 future events on three Microsoft Excel spreadsheet templates: 20 negative events (i.e., fears and worries), 20 positive events (i.e., hopes and dreams), and 36 neutral events (i.e., routines and mundane hypothetical scenarios). Participants completed four cells for each event: (i) a tagline in less than eight words describing the essence of the event, (ii) a more elaborate description of details that the experimenter used to verify the event’s compliance with the rules, (iii) a Cue Word, an obvious reminder that was used to evoke the event during training, and (iv) a Key Detail, a single word expressing a central event detail not mentioned in the tagline and obvious only to the participant, which was used to assess event recall during the immediate and delayed tests.

Although all participants generated the same amount and types of events at the outset, we drew selectively from these events to construct the stimuli set that differed in the four groups. Specifically, we used only 40 of the 76 events generated initially as stimuli for each participant (32 critical events for the training, 4 events for practice, and 4 events for context reinstatement during the recall test). The reasons why people generated more material than we used for their training were that we (i) wanted to be certain that all participants generated an equal number of events in the negative, neutral, and positive conditions (avoiding any biases) and (ii) the INI task for each of our four groups required materials with different valences. Thus, we selected a subset of critical events according to the condition to which participants had been assigned. When we assigned participants to suppress negative events and imagine positive events, we set neutral events aside and used only the positive and negative events. When we assigned them to suppress negative and imagine neutral events, we set aside positive events and used negative events and neutral events. When we assigned them to suppress neutral events and imagine positive events, we set aside negative events, selecting positive and neutral events, and when we assigned participants to suppress and imagine neutral events, we set aside all positive and negative events, focusing exclusively on neutral events. For each participant, we allocated eight events to each of four conditions: Imagine, No-Imagine, Baseline Imagine, and Baseline No-Imagine. For example, in the Suppress-Negative, Imagine-Positive group, Imagine and Baseline Imagine would each be composed of eight positive events, and No-Imagine and Baseline No-Imagine would be composed of eight negative events. Within a condition pair (e.g., Imagine and Baseline Imagine or No-Imagine and Baseline No-Imagine), we assigned events to conditions algorithmically to match a given manipulation with its baseline as closely as possible. For example, through an automated matching algorithm, we maximized the average similarity of six ratings of event characteristics (mood, vividness, likelihood of occurrence, distance in the future, frequency of thought, and degree of current concern; see the “Procedure” section for details) across the No-Imagine and Baseline No-Imagine conditions and between the Imagine and Baseline Imagine conditions.

We administered mental health questionnaires either once or multiple times (pretraining, posttraining, and follow-up) using the secure online survey tool Qualtrics (*71*). We measured our six target mental health indices at all three time points and included both positive and negative mental health indices. The positive indices were Warwick-Edinburgh Mental Well-being Scale (WEMWBS) (*72*) and the positive component of the Positive and Negative Affect Schedule (PANAS) (*73*). The negative indices were State portion of the State-Trait Anxiety Inventory Form Y-1 (STAI-State) (*74*), Penn State Worry Questionnaire–Past Day (PSWQ-PD) (*75*), Beck’s Depression Inventory II (BDI) (*76*), and the negative component of the Positive and Negative Affect Schedule (PANAS) (*73*). We modified the BDI to exclude a single item referring to suicidal thoughts because of ethical concerns about triggering suicidal ideation. The positive and negative indices are computed simply by averaging over the POMP scores separately for the positive and negative mental health scales. Given our focus on posttraumatic stress and anxiety during the pandemic and given a priori concerns about ironic mental health effects, we measured pandemic-related PTSD and trait anxiety to scrutinize our intervention’s effect on these vulnerable populations, via the Impact of Events Scale–Revised (IES) (*77*) and the trait portion of the State-Trait Anxiety Inventory Form Y-1 (STAI-Trait) (*74*). IES was administered before training and at follow-up to estimate dynamic changes in pandemic-related circumstances over the 3-month delay, which was then incorporated in the latent growth curve modeling (LGCM) analysis (see Fig. 5). Last, we included exploratory scales, the results of which will not be reported in this paper, but the data are uploaded to Dataverse (*78*), including the Optimism Pessimism Instrument (OPI) (*79*), Health Anxiety Inventory (HAI-18) (*80*), Intolerance of Uncertainty Scale (IUS-12) (*81*), Five Facets of Mindfulness (FFMQ) (*82*), Cognitive Flexibility Inventory (CFI) (*83*), Meaning in Life Questionnaire (MIL) (*84*), Metacognitive belief questionnaire (MCQ-30) (*85*), and Short UPPS-P Impulsive Behavior Scale (SUPPS-P) (*86*).

### Procedure

The experiment includes a set of initial training sessions and a follow-up session 3 months later. Here, we describe the procedures of these two segments. The initial stage of the study was conducted in three phases conducted over 5 days: introduction, training, and testing (see Fig. 1C). In the introductory session (day 1), we briefly introduced the study, collected verbal consent, administered preexperimental questionnaires, and then provided instructions for the event generation task. The event generation phase (days 1 to 2) consisted of two tasks: listing events and then rating them on several scales. These two tasks were repeated three times, once for each event valence category (negative, neutral, and positive). We emailed the event generation spreadsheet template to participants for a given valence (e.g., negative), and once returned and completed, the next event generation spreadsheet would be sent, and this would continue until all three valence categories (76 events) had been completed. We randomized the order in which the negative, neutral, and positive events were generated for each participant.

Each event needed to comply with six rules that were clearly explained to participants. Specifically, events needed to (i) be genuinely positive (i.e., a future hope that brings incredible joy), negative (i.e., a feared future event that has caused and continues to cause worry), or neutral (i.e., routines and mundane tasks); (ii) be something that the participant can and has vividly imagined from their first-person perspective; (iii) concern possible developments in the participant’s life that could take place within the next 2 years; (iv) be a specific episode with a particular time and place; (v) only last between a few minutes to 1 day; and (vi) be current concerns or recurrent fears (i.e. thought of at least three times in the past 6 months) in the case of negative events. Upon completion of each template, the experimenter checked to ensure that (i) each event followed the six rules, (ii) no repeats of words are present for Cue Word and Key Detail across all templates, and (iii) Key Detail words do not appear in the tag lines and gave feedback by email if any edits were required. Once all events complied with the rules, the experimenter emailed the participant the ratings task in html generated via PsyToolkit (*87*), which consisted of eight ratings per event: vividness, likelihood of occurrence, distance in the future, level of anxiety about the event (for positive events: joy), frequency of thought, degree of current concern, long-term impact, and emotional intensity. The ratings were all rated on a five-point scale, except for emotional intensity, which is on a nine-point scale (see table S1C for event characteristics and see the notes of this table for details on each of the rating scales). Note that the anxiety rating (on a five-point scale) for each event was a core “event”–dependent variable measured before and after training (and at follow-up) (Fig. 1), whereas emotional intensity (on a nine-point scale) was used as a distinct one-time pretraining predictor measure (see, e.g., Fig. 2, where participants are sorted by this measure). The same sequenced procedure, event generation, experimenter checks, and participant ratings repeated for the next two templates during the time between the introduction session and the first day of training.

After participants completed the event generation phase, they proceeded to the first day of training (day 3; see Fig. 1C). The first day consisted of a brief questionnaire (given daily during training), a criterion test, practice at the suppression task on filler items, and lastly the INI training. The brief daily questionnaire recorded the following: (i) occurrence of any mood-altering experiences since the day before, (ii) overall mood and arousal based on Self-Assessment Manikin (*88*), (iii) hours of sleep on the preceding night (1 to 11 scale), and (iv) degree of tiredness (1 to 10).

The main experimental tasks then ensued. During these tasks, all stimuli were presented via screen sharing during videoconferencing from scripts written in MATLAB with Psychophysics Toolbox extensions (*89*, *90*). We began with a criterion test that ensured that participants could remember the associations between their Cue Word and Key Detail for each of the 40 events before suppression training. For each event, we presented the Cue Word and tested whether the participant could remember the Key Detail, after which we gave feedback about the correct Key Detail that they had originally listed. During this test, we required three verbal responses for each item, when presented with its Cue Word (600-ms interstimulus interval): whether (i) they could silently remember the personal event associated with the Cue Word (yes/no, 5 s), (ii) they could recall the Key Detail by saying it aloud (5 s), and (iii) the event that they were thinking of in response to the Cue Word during the first “yes/no” response was the right event (3 s), given the feedback provided (2 s). To calculate the accuracy of participants’ responses, the experimenter coded each trial upon hearing the three verbal responses. If the participant gave no response, then that event was coded as incorrect; if they responded, then we considered an event was accurately recalled if the participant stated that they could recall the event (yes/no), recalled the correct Key Detail, and confirmed that the Key Detail went with the event that they had recalled. We repeated the criterion test up to three times to ensure that participants achieved above 90% accuracy. We displayed events in the criterion test such that no more than two consecutive trials were from the same condition. On the first test, we picked events randomly from the condition set without replacement (aside from the aforementioned constraint on condition repetition) and followed the same order for the repeated criterion tests.

Following the criterion test, participants practiced the INI task and then underwent the training. Each trial first showed a fixation cross for 500 ms followed by a Cue Word for 4 s [600-ms inter-stimulus interval (isi)] in either green or red font depending on whether it was an Imagine or No-Imagine trial, respectively. For Imagine trials, we instructed participants to do the following for as long as the green Cue Word remained on the screen: (i) Read the Cue Word silently and recognize the event to which it referred. (ii) Recall the associated Key Detail to mind silently. (iii) Imagine the future event in vivid detail. (iv) Add a novel feature to the event each time the Cue Word repeated. For the two groups that imagined positive events on Imagine trials, we further asked them to create a positive and hopeful feeling each time they imagined the event. For No-Imagine trials, we instructed participants to do the following for as long as the red Cue Word remained on the screen: (i) Read the Cue Word silently and recognize the event to which it referred. (ii) Stop any further imagination of the event by blocking the event and any associated details out and keeping them out of mind. To ensure that participants engaged direct suppression and not thought substitution (*91*), the experimenter urged participants not to replace the unwanted thoughts (such as the Key Detail or other specifics of the event) with something else and to instead remain focused on the Cue Word and keep their mind blank during the No-Imagine trials. During the practice phase, we showed four filler events twice, across two blocks (i.e., eight trials in total). We presented Cue Words in a pseudorandom order such that the Cue Word was randomly selected from the set of items in its condition; however, the Cue Word was repeated only after all of the other Cue Words in the same condition had been shown. Following the practice phase, we verbally administered a diagnostic questionnaire that allowed the experimenter to give constructive feedback to ensure that all instructions for the INI task were followed correctly.

The training phase followed the same procedure as the practice phase. During each of the four training runs, we repeated 16 events (i.e., 8 Imagine and 8 No-Imagine) three times, yielding 192 trials. Thus, across the whole session, the eight Imagine and eight No-Imagine Cue Words repeated 12 times. We determined the random ordering of conditions (I or NI) over trials once at the study’s outset, ensuring that no more than two consecutive trials came from the same condition; all participants followed this order. Cue Words were presented in a pseudorandom order across the blocks following the same rule as the practice phase. In between runs, we provided a short (up to 60 s) break for the participant to rest their eyes. Diagnostic questionnaire was administered again verbally after the second block (i.e., halfway through the training). Trial timings matched the timings in the practice phase: fixation cross for 5 s and colored Cue Word for 4 s, with 600-sms isi.

At the beginning of the training phase, the experimenter always turned off her video camera, whereas the participants kept their cameras on. After a training session and before ending the activities on the first day, we told participants to not intentionally engage with the materials outside of the training sessions (i.e., to not rehearse the word pairs). On the second and third training days, participants followed the same procedure, except that we omitted the criterion test. We conducted the three training days consecutively.

After the INI task on the final training day, we tested participants on all 40 of the main target events in the experiment. The testing phase comprised of (i) a recall test in which a correct verbal response of the corresponding Key Detail to the Cue Word presented constituted an accurate response and (ii) a rating task in which vividness and affect ratings were given through html files sent by the experimenter for each of the events. During these tests, events were ordered such that no more than two consecutive trials were drawn from the same condition. After the tests, we included a questionnaire probing participants’ out-of-experiment activities, as well as a series of mental health questionnaires.

For the follow-up session, we contacted participants via email to schedule the follow-up session 3 months following the date of their introduction session (±1 week). The follow-up session lasted around 1 hour and consisted of a recall test, vividness and affect ratings, and follow-up questionnaires. The Key Word recall test was the exact same one as was used in the testing phase on the last day of training, with only the time of stimulus display extended from 4 to 5 s.

### Statistical analyses

The accuracy reported for the final key detail recall was not conditionalized on the criterion test from the first day of training. To put mental health questionnaires on a uniform scale, all scores included in this paper were converted (unless otherwise stated as raw data) to POMP scores, which are calculated as POMP = 100 ∗ (raw − min)/(max − min). Analyses of repeated measure analyses of variance (ANOVAs), analyses of covariance (ANCOVAs), and *t* tests were performed using JASP (*92*). Component values from PCAs were derived from IBM SPSS Statistics 27 (*93*). Statistical analyses of structural equation modeling were carried out in R using the Lavaan package (*94*) for a latent growth curve analysis of the questionnaire scores collected across the three time points (pre, post, and follow-up). Robust correlation analyses were conducted in R using a script that automatically gives an output for the most appropriate of the three correlation methods (Pearson’s, Spearman skipped, percentage bend) upon inspection for outliers and normality (*95*).
