# Supplementary material for: Improving mental health by training the suppression of unwanted thoughts
Source: Sci Adv. 2023 Sep 20;9(38):eadh5292. doi: 10.1126/sciadv.adh5292 (PMC10511195; doi:10.1126/sciadv.adh5292)
Supplement: Supplementary file 1 — Figs. S1 to S4 Tables S1 to S12 [file sciadv.adh5292_sm.pdf]

Science Advances
AAAS

Supplementary Materials for

Improving mental health by training the suppression of unwanted thoughts

Zulkayda Mamat and Michael C. Anderson

Corresponding author: Michael C. Anderson, michael.anderson@mrc-cbu.cam.ac.uk

Sci. Adv. **9**, eadh5292 (2023)
DOI: 10.1126/sciadv.adh5292

This PDF file includes:

Figs. S1 to S4
Tables S1 to S12

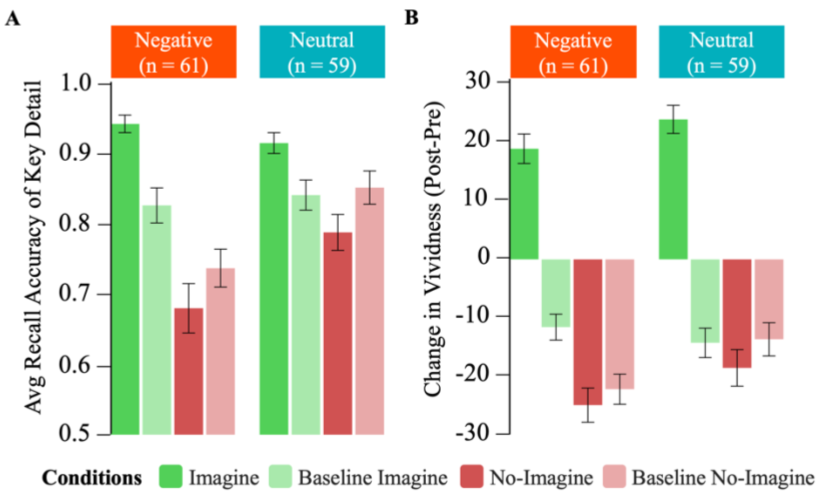

Fig. S1. Impact of imagination and suppression on event memory. (**A**) Imagining future events increased the probability of recalling events' key details (dark green bars), relative to the probability of recalling the key details in matched Baseline events that did not receive repeated imagination trials during the 3-days of training (light green bars). In contrast, suppressing thoughts of future events reduced the probability of recalling events' key details (dark red bars) relative to the probability of recalling key details for matched Baseline events (light red bars). Half of the participants suppressed feared events (left half), and half suppressed neutral events (right half). Notably, within the Negative group (left half), the events in the No-Imagine and Baseline-No-Imagine conditions are uniformly negative, whereas within the neutral group (right half), those same conditions concerned uniformly neutral events. In contrast, within the Negative group (left half), half of the participants imagined Positive events and half, Neutral events (with corresponding Positive and Neutral Baseline-Imagine events); in the Neutral group, the same was true (see Fig. 1B for a design overview). (**B**) The increase in the vividness of events post training, compared to pre-training was greater when participants repeatedly imagined those events during training (dark green bars) compared to the vividness changes observed for Baseline events that were not repeatedly imagined during training (light green bars). In contrast, the reduction in vividness of events post-training, compared to pre-training was greater when participants repeatedly suppressed those events during training (dark red bars), compared to changes observed for Baseline events that were not repeatedly suppressed (light red bars). The organization of Panel B is analogous to Panel A, with half of the participants suppressing Negative events (left half) and half suppressing Neutral events (right half). Instead of plotting changes in the vividness rating scale directly, scores in Panel B are computed as POMP scores (percentage of maximum point; see section on *Statistical Analyses*). Error bars reflect standard errors.

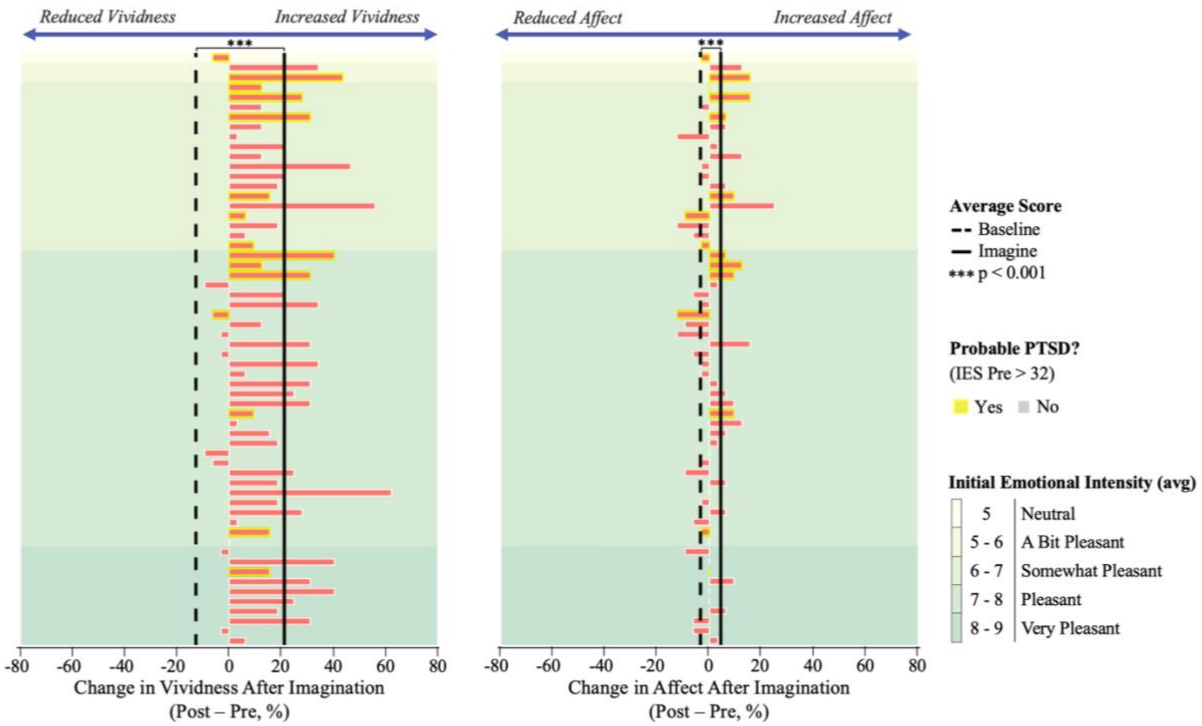

Fig. S2. Impact of imagination of vividness and affect for hopeful events. Imagining hopeful events increased their vividness on an immediate test for nearly all participants, irrespective of the initial emotional intensity of those hopes and post-traumatic stress status; horizontal bars are individual participants' average change in vividness (post – pre, POMP scores). Participants are sorted by the average rated emotional intensity of their hopes pre-training from most pleasant (bottom bars, darker green background) to neutral (top bars, yellower background); yellow highlights on bars indicate participants with probable PTSD. Vividness facilitations are significantly greater for Imagine than for Baseline items (mean changes indicated by solid black vs dotted vertical bars). In the Imagine-Positive group, imagination increased positive affect for the imagined hopes, as seen by the greater increase in affect (post – pre) for imagined events (solid black bar) compared to baseline events (dotted bar).

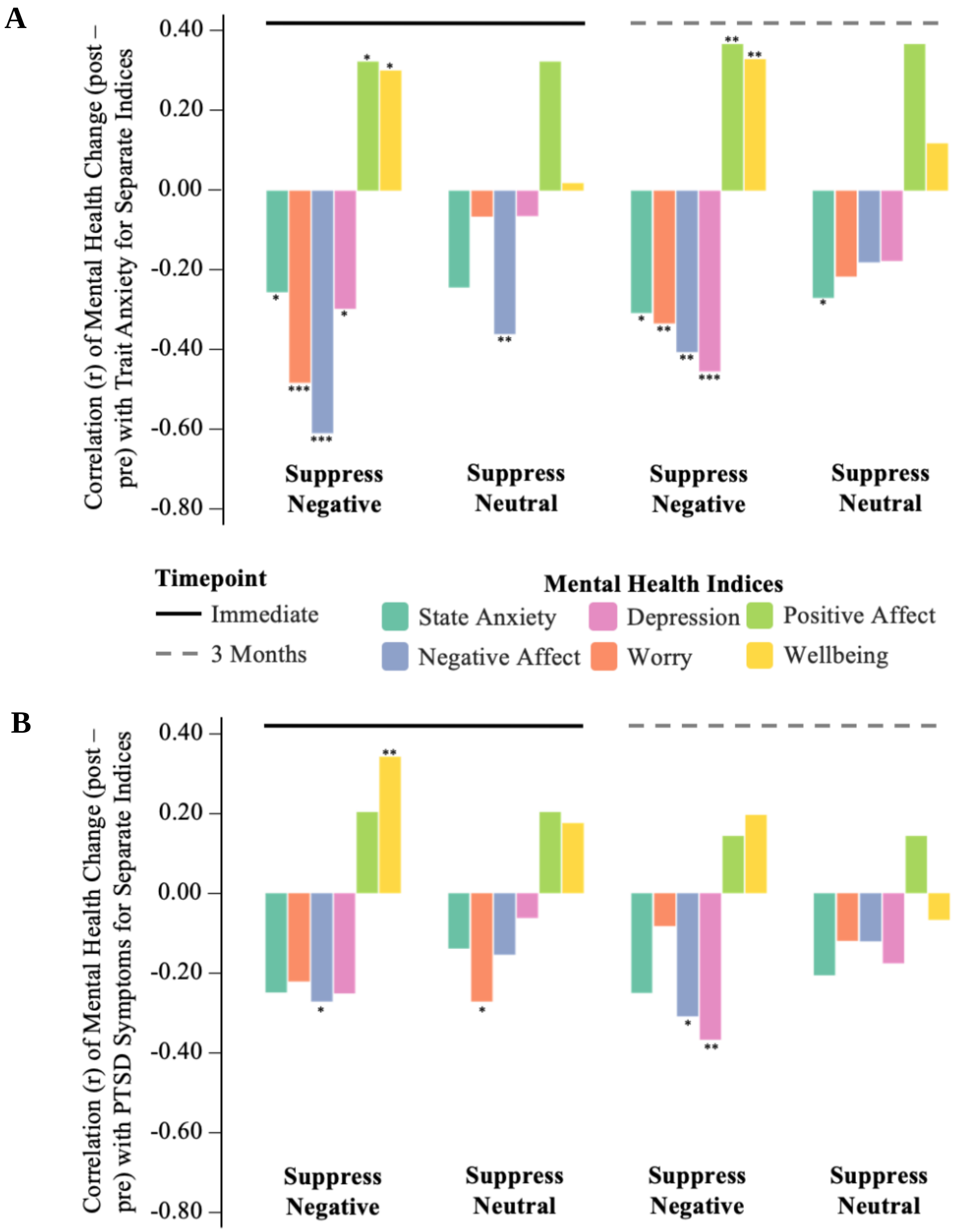

Fig. S3. Pre-Training Trait Anxiety and Post-Traumatic Stress status predicted improvement on individual mental-health measures, but mainly when participants suppressed fears and not neutral events. (**A**) Correlations (r values) are plotted on the y-axis; significance is indicated by asterixis (*** < 0.001; ** < 0.01; * < 0.05). Trait anxiety levels prior to training predicted whether participants showed significant improvements on negative mental health indices (extending downward) and positive indices (extending upwards) arising on the immediate test (post – pre; bars below solid lines) and the 3-month follow-up (follow-up – pre; bars below dotted lines). Trait anxiety predicted improvement (i.e., for all six measures of mental health state) for those who suppressed fears (Suppress-Negative), more so than for those who suppressed neutral events (Suppress-Neutral). (**B**) Post-traumatic stress scores predicted

improvement (post – pre) on some negative mental health indices (negative affect and depression) and positive indices (wellbeing) on the immediate test (bars below solid lines) and the 3-month follow-up (follow-up – pre; bars below dotted lines). Such correlations were significant after suppressing fears (Suppress-Negative), but generally not after suppressing neutral events (Suppress-Neutral).

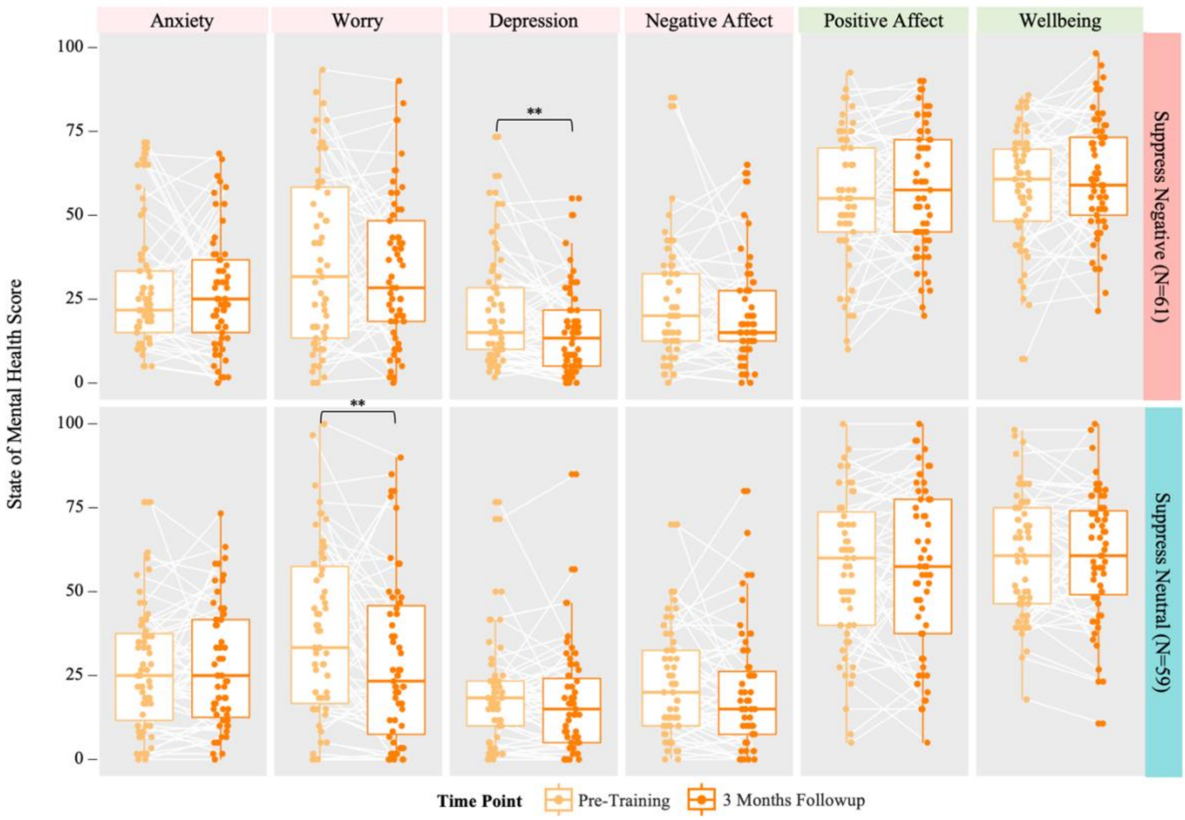

Fig. S4. Mental health changes after a 3-month delay in our whole sample. Training at suppressing fears (upper half, red label) significantly reduced depression after 3 months compared to pre-training; training at suppressing neutral events (lower half, blue label) reduced worry after 3 months. Individual participants are indicated by dots connected by white lines; boxes reflect interquartile range, and lines reflect median scores.

Table S1.

A. Demographic Characteristics of Participants

Demographic Characteristic	Suppress Negative		Suppress Neutral		Sig.
	n	%	n	%	
**Gender**					
Female	51	83.61	42	71.19	-
Male	10	16.39	14	23.73	-
Missing	0	0.00	3	5.09	-
**Ethnicity**					
Any other White background	21	34.43	11	18.64	-
Any other ethnic group	0	0.00	1	1.69	-
Arab	1	1.64	0	0.00	-
Asian / Asian British	7	11.48	11	18.64	-
Black / African / Caribbean / Black British	2	3.28	6	10.17	-
Mixed / Multiple ethnic background	3	4.92	3	5.08	-
White British	27	44.26	25	42.37	-
Missing	0	0.00	2	3.39	-

Note. N = 120. Participants were on average 27.41 years old (*SD* = 10.21), and participant age did not differ by group.

B. Participants' Mental Health Characteristics Prior to Training

Baseline characteristic	Suppress Negative		Suppress Neutral		Sig.
	μ	SD	μ	SD	
**Positive Mental Health Indices**					
Positive Negative Affect Schedule - Positive	31.93	7.80	32.58	8.89	ns
Warwick-Edinburgh Mental Well-being Scale	46.87	9.52	48.00	10.30	ns
**Negative Mental Health Indices**					
State Trait Anxiety Inventory - State	36.43	10.74	36.31	11.15	ns
Penn State Worry Questionnaire	21.54	15.53	22.75	15.28	ns
Positive Negative Affect Schedule - Negative	19.43	6.97	18.97	6.59	ns
Beck's Depression Inventory II	12.54	9.79	11.34	9.29	ns
**Predictors of Training Response**					
State Trait Anxiety Inventory - Trait	42.72	12.18	43.44	12.65	ns
Impact of Events Scale Revised	22.87	14.69	24.17	17.37	ns

Note. Positive and Negative Mental Health Indices contributed to the main indices of mental health and were also measured post-training and at followup. Here we display raw questionnaire scores, though in most figures and analyses we used POMP scores. Significance indicated is from independent t-test between Suppress Negative and Suppress Neutral groups. Non-significance is indicated by "ns".

C. Event Characteristics

To-Be Suppressed Events	**Suppress Negative** Baseline		No-Imagine		**Suppress Neutral** Baseline		No-Imagine	
	µ	SD	µ	SD	µ	SD	µ	SD
Current Source of Concern	3.17	0.69	3.13	0.67	2.34	1.02	2.37	1.11
Distance in the Future	2.77	0.59	2.85	0.70	1.94	0.60	1.88	0.65
Emotional Intensity	2.56	0.75	2.53	0.83	5.52	0.57	5.52	0.69
Frequency of Thought	3.09	0.66	3.00	0.61	2.48	0.88	2.44	0.95
Likelihood of Occurrence	2.73	0.55	2.71	0.60	4.19	0.58	4.20	0.61
Long-term Impact	3.54	0.55	3.49	0.65	2.28	0.76	2.23	0.84

To-Be Imagined Events	**Imagine Positive** Baseline		Imagine		**Imagine Neutral** Baseline		Imagine	
	µ	SD	µ	SD	µ	SD	µ	SD
Current Source of Concern	3.17	0.75	3.20	0.75	2.25	0.89	2.28	0.91
Distance in the Future	3.26	0.67	3.27	0.71	1.70	0.50	1.80	0.49
Emotional Intensity	7.43	0.73	7.37	0.78	5.58	0.73	5.54	0.68
Frequency of Thought	3.15	0.60	3.15	0.65	2.43	0.84	2.43	0.85
Likelihood of Occurrence	3.54	0.65	3.38	0.71	4.30	0.55	4.23	0.52
Long-term Impact	3.55	0.60	3.51	0.62	2.23	0.87	2.25	0.87

Note. All reported event rating measures were collected prior to suppression training and were used algorithmically to assign events so as to match Baseline and Imagine or Baseline and No-Image conditions prior to training. **Current Source of Concern**: the extent to which the event is a source of current focus of one's thoughts, where 1 is not at all, 3 is to some extent, and 5 is most definitely. **Distance in Future**: how far in the future one imagines the event happening, with 1 being very soon and 5 being further in the future, with the maximum being 2 years away. **Emotional Intensity**: how strongly the event makes one feel, where 1 is very unpleasant and 9 is very pleasant (Manikin scale wherein 5 is neutral). **Frequency of Thought**: how often one tends to think about the event, with 1 being rarely and 5 being very often. **Likelihood of Occurrence**: how likely the event is to actually happen to the person, with 1 being a very unlikely event and 5 being almost certain to happen. **Long-Term Impact**: how much long-term impact (aversive or beneficial) the event will have on one's physical and/or psychological wellbeing if the event were to come true, such that: 1-none at all, 2-negligible, 3-small, 4-moderate, 5-large/formidable. Significance indicated by independent t-test between the *Baseline* and the manipulation condition (*No-Imagine* or *Imagine*). No significant differences were found between Baseline and No-Imagine (or Baseline and Imagine) conditions in any of the rating scales for any group.

Table S2.

Immediate Effects of Suppression & Imagination on Key Detail Recall and Vividness

	**Suppression**						**Imagination**					
	Baseline		No-Imagine		SIF		Baseline		Imagine		IIF	
	μ	SD	μ	SD	μ	SD	μ	SD	μ	SD	μ	SD
**Key Detail Recall**												
Suppress Negative	0.74	0.21	0.68	0.27	0.06	0.26	0.83	0.19	0.94	0.10	0.12	0.19
Suppress Neutral	0.85	0.18	0.79	0.20	0.06	0.19	0.84	0.16	0.91	0.11	0.07	0.17
Overall	0.79	0.20	0.73	0.24	0.06	0.23	0.83	0.18	0.93	0.11	0.10	0.18
**Vividness**												
Suppress Negative	-0.88	0.80	-0.99	0.91	0.11	0.60	-0.46	0.69	0.75	0.78	1.21	1.07
Suppress Neutral	-0.54	0.87	-0.74	0.95	0.19	0.68	-0.57	0.77	0.95	0.73	1.52	1.04
Overall	-0.72	0.85	-0.87	0.94	0.15	0.64	-0.51	0.73	0.85	0.76	1.36	1.06

Note. SIF: suppression induced forgetting, IIF: imagination induced facilitation. Key Detail recall reflects the percentage of items correctly recalled. Vividness reflects the change in rated vividness after suppression compared to before it (post – pre).

Table S3.

A. *Relative Risk of Increased Accessibility (Reversals) after Training (post – pre) for Suppressed Events Compared to Baseline Events in the Suppress-Negative and Suppress-Neutral groups.*

	**Suppress Negative**			**Suppress Neutral**		
	# Subj With Reversals	# Subj Without Reversals	Absolute Risk (%)	# Subj With Reversals	# Subj Without Reversals	Absolute Risk (%)
**Key Detail Recall**						
No-Imagine	1.00	60.00	1.64	0.00	59.00	0.00
Baseline No-Imagine	1.00	60.00	1.64	4.00	55.00	6.78
Relative Risk [95% CI]	1.00 [0.06, 15.63]			Undefined		
**Vividness**						
No-Imagine	6.00	55.00	9.84	15.00	44.00	25.42
Baseline No-Imagine	5.00	56.00	8.20	14.00	45.00	23.73
Relative Risk [95% CI]	1.20 [0.39, 3.72]			1.07 [0.57, 2.02]		

Note. Relative risks are “undefined” if a calculation resulted in division by zero. If the relative risk = 1, or if the 95% confidence interval includes 1, then there is no significant difference between the groups.

B. *Average Number of Fears Per Participant that Show Increased Accessibility (Reversals) After Training Compared to Before (post – pre) for the No-Imagine and Baseline Conditions, separately for the Suppress-Negative and Suppress-Neutral Groups. Reversals Shown for Both Key Detail Recall and Vividness.*

	**Suppress Negative**		**Suppress Neutral**	
	μ	SD	μ	SD
**Key Detail Recall**				
No-Imagine	0.02	0.13	0.00	0.00
Baseline No-Imagine	0.02	0.13	0.07	0.25
**Vividness**				
No-Imagine	0.10	0.30	0.25	0.44
Baseline No-Imagine	0.08	0.28	0.24	0.43

Table S4.

Delayed Effects of Suppression & Imagination on Key Detail Recall and Vividness at 3 Months

	**Suppression**						**Imagination**					
	Baseline		No-Imagine		SIF		Baseline		Imagine		IIF	
	μ	SD	μ	SD	μ	SD	μ	SD	μ	SD	μ	SD
**Key Detail Recall**												
Suppress Negative	0.17	0.21	0.18	0.20	0.00	0.19	0.21	0.20	0.25	0.19	0.04	0.20
Suppress Neutral	0.19	0.17	0.18	0.18	0.00	0.20	0.25	0.21	0.31	0.22	0.06	0.22
Overall	0.18	0.19	0.18	0.19	0.00	0.19	0.23	0.21	0.28	0.20	0.05	0.21
**Vividness**												
Suppress Negative	-0.61	0.84	-0.57	0.76	-0.04	0.66	-0.31	0.75	-0.12	0.75	0.19	0.75
Suppress Neutral	-0.22	0.78	-0.18	0.80	-0.04	0.65	-0.16	0.64	0.05	0.73	0.22	0.66
Overall	-0.42	0.83	-0.38	0.80	-0.04	0.65	-0.24	0.70	-0.03	0.74	0.20	0.71

Note. SIF: suppression induced forgetting, IIF: imagination induced facilitation. Key Detail recall reflects the percentage of items correctly recalled. Vividness reflects the change in rated vividness after suppression compared to before it (post – pre).

Table S5.

A. *Relative Risk of Increased Accessibility (Reversals) after Training (followup – pre) for Suppressed Events Compared to Baseline Events in the Suppress-Negative and Suppress-Neutral groups.*

	**Suppress Negative**			**Suppress Neutral**		
	# Subj With Reversals	# Subj Without Reversals	Absolute Risk (%)	# Subj With Reversals	# Subj Without Reversals	Absolute Risk (%)
**Key Detail Recall**						
No-Imagine	0.00	61.00	0.00	0.00	59.00	0.00
Baseline No-Imagine	0.00	61.00	0.00	0.00	59.00	0.00
Relative Risk [95% CI]		Undefined			Undefined	
**Vividness**						
No-Imagine	14.00	47.00	22.95	21.00	38.00	35.59
Baseline No-Imagine	14.00	47.00	22.95	26.00	33.00	44.07
Relative Risk [95% CI]		1.00 [0.52, 1.92]			0.81 [0.52, 1.26]	

Note. Relative risks are "undefined" if a calculation resulted in division by zero. If the relative risk = 1, or if the 95% confidence interval includes 1, then there is no significant difference between the groups.

B. *Average Number of Fears Per Participant that Show Increased Accessibility (Reversals) After Training Compared to Before (followup – pre) for the No-Imagine and Baseline Conditions, separately for the Suppress-Negative and Suppress-Neutral Groups. Reversals Shown for Both Key Detail Recall and Vividness.*

	**Suppress Negative**		**Suppress Neutral**	
	μ	SD	μ	SD
**Key Detail Recall**				
No-Imagine	0.00	0.00	0.00	0.00
Baseline No-Imagine	0.00	0.00	0.00	0.00
**Vividness**				
No-Imagine	0.23	0.42	0.36	0.48
Baseline No-Imagine	0.23	0.42	0.44	0.50

Table S6.

Effects of Suppression & Imagination on Subjective Affect

	**Suppression**						**Imagination**					
	Baseline		No-Imagine		SIF		Baseline		Imagine		IIF	
	µ	SD	µ	SD	µ	SD	µ	SD	µ	SD	µ	SD
**Immediate (Post - Pre)**												
Suppress Negative	-0.34	0.68	-0.45	0.74	0.11	0.51	-0.19	0.48	0.07	0.57	0.26	0.55
Suppress Neutral	-0.25	0.75	-0.33	0.65	0.09	0.55	-0.09	0.54	0.28	0.57	0.37	0.52
Overall	-0.29	0.71	-0.39	0.70	0.10	0.53	-0.14	0.51	0.17	0.58	0.31	0.53
**Delayed (Followup - Pre)**												
Suppress Negative	-0.59	0.73	-0.57	0.70	-0.01	0.56	-0.29	0.57	-0.20	0.56	0.09	0.47
Suppress Neutral	-0.17	0.68	-0.17	0.72	0.00	0.58	0.00	0.73	-0.01	0.63	0.00	0.53
Overall	-0.38	0.73	-0.37	0.73	0.00	0.57	-0.15	0.67	-0.11	0.60	0.04	0.50

Note. SIF: suppression induced forgetting, IIF: imagination induced facilitation. Scores for subjective affect reflect the change in affective rating after suppression (either Post – Pre in the Immediate condition, or Followup – Pre in the Delayed condition).

Table S7.

Relative Risk of Increased Affect (Reversals) after Training (Post-Pre or Followup – Pre) for Suppressed Events Compared to Baseline Events in the Suppress-Negative and Suppress-Neutral Groups.

	**Suppress Negative**			**Suppress Neutral**		
	# Subj With Reversals	# Subj Without Reversals	Absolute Risk (%)	# Subj With Reversals	# Subj Without Reversals	Absolute Risk (%)
**Immediate (Post - Pre)**						
No-Imagine	15.00	46.00	24.59	13.00	46.00	22.03
Baseline No-Imagine	15.00	46.00	24.59	15.00	44.00	25.42
Relative Risk [95% CI]	1.00 [0.54, 1.86]			0.87 [0.45, 1.66]		
**Delayed (Followup - Pre)**						
No-Imagine	12.00	49.00	19.67	24.00	35.00	40.68
Baseline No-Imagine	10.00	51.00	16.39	19.00	40.00	32.20
Relative Risk [95% CI]	1.20 [0.56, 2.57]			1.26 [0.78, 2.04]		

Table S8.

Summary of Relative Risks, Based on Calculations in Detailed Table Below. Significant reductions in risk of ironic worsening were observed for depression and well-being on the immediate test (bold).

	Immediate	Delayed
State Trait Anxiety Inventory – State	0.79 [0.52, 1.21]	1.25 [0.84, 1.85]
Penn State Worry Questionnaire	1.19 [0.63, 2.25]	1.83 [1.17, 2.85]
Positive Negative Affect Schedule - Negative	0.74 [0.40, 1.38]	0.85 [0.54, 1.33]
Beck's Depression Inventory II	**0.43 [0.23, 0.78]**	0.67 [0.40, 1.14]
Positive Negative Affect Schedule - Positive	0.76 [0.52, 1.11]	0.93 [0.64, 1.36]
Warwick-Edinburgh Mental Well-being Scale	**0.56 [0.33, 0.94]**	0.68 [0.47, 1.00]

Relative Risk of Ironic Worsening (Reversals) in Mental Health After Training the Suppression of Fears, Relative to Training the Suppression of Neutral Events

	Immediate			Delayed		
	# Subj With Reversals	# Subj Without Reversals	Absolute Risk (%)	# Subj With Reversals	# Subj Without Reversals	Absolute Risk (%)
**State Trait Anxiety Inventory – State**						
No-Imagine	23.00	38.00	37.70	31.00	30.00	50.82
Baseline No-Imagine	28.00	31.00	47.46	24.00	35.00	40.68
Relative Risk [95% CI]		0.79 [0.52, 1.21]			1.25 [0.84, 1.85]	
**Penn State Worry Questionnaire**						
No-Imagine	16.00	45.00	26.23	34.00	27.00	55.74
Baseline No-Imagine	13.00	46.00	22.03	18.00	41.00	30.51
Relative Risk [95% CI]		1.19 [0.63, 2.25]			1.83 [1.17, 2.85]	
**Positive Negative Affect Schedule - Negative**						
No-Imagine	13.00	48.00	21.31	22.00	39.00	36.07
Baseline No-Imagine	17.00	42.00	28.81	25.00	34.00	42.37
Relative Risk [95% CI]		0.74 [0.40, 1.38]			0.85 [0.54, 1.33]	
**Beck's Depression Inventory II**						
No-Imagine	11.00	50.00	18.03	16.00	45.00	26.23
Baseline No-Imagine	25.00	34.00	42.37	23.00	36.00	38.98
Relative Risk [95% CI]		0.43 [0.23, 0.78]			0.67 [0.40, 1.14]	
**Positive Negative Affect Schedule - Positive**						
No-Imagine	25.00	36.00	40.98	28.00	33.00	45.90

Baseline No-Imagine	32.00	27.00	54.24	29.00	30.00	49.15
Relative Risk [95% CI]	0.76 [0.52, 1.11]			0.93 [0.64, 1.36]		
**Warwick-Edinburgh Mental Well-being Scale**						
No-Imagine	15.00	46.00	24.59	24.00	37.00	39.34
Baseline No-Imagine	26.00	33.00	44.07	34.00	25.00	57.63
Relative Risk [95% CI]	0.56 [0.33, 0.94]			0.68 [0.47, 1.00]		

Table S9.

A. Training Benefits on our Immediate Test for Participants with High Trait Anxiety.

Mental Health Questionnaires	**Suppress Negative**		Sig. *Time*	**Suppress Neutral**		Sig. *Time*	Sig. *Group*
	μ	SD		μ	SD		
State Trait Anxiety Inventory - State	-4.93	20.96	ns	-2.74	13.69	ns	ns
Penn State Worry Questionnaire	-18.19	26.77	**	-8.27	18.45	*	ns
Positive Negative Affect Schedule - Negative	-15.33	16.59	***	-8.57	13.68	**	ns
Beck's Depression Inventory II	-8.12	13.48	**	0.00	13.12	ns	*
Positive Negative Affect Schedule - Positive	7.72	13.52	*	-2.86	14.67	ns	*
Warwick-Edinburgh Mental Well-being Scale	7.30	9.95	**	-0.26	9.51	ns	**

Note. Participants are included as having high anxiety if they scored > 44 on the pre-training baseline measure of the Trait portion of the State Trait Anxiety Inventory. Scores reflect the change in mental health state after training relative to baseline measures prior to training (post – pre, POMP scores). Significance of Time reflects a paired samples student t-test between pre and post training. Significance of Group is derived from independent samples student t-tests between Suppress-Negative and Suppress-Neutral groups on the post - pre values. The p-value is indicated as such: < 0.001(***), < 0.01(**), < 0.05(*), <= 0.1(†), > 0.1(ns).

B. Training Benefits on our Immediate Test for Participants with Likely PTSD

Mental Health Questionnaires	**Suppress Negative**		Sig. *Time*	**Suppress Neutral**		Sig. *Time*	Sig. *Group*
	μ	SD		μ	SD		
State Trait Anxiety Inventory - State	-10.61	20.59	†	-1.02	17.73	ns	ns
Penn State Worry Questionnaire	-19.24	25.78	*	-14.91	22.96	*	ns
Positive Negative Affect Schedule - Negative	-12.95	23.90	*	-6.67	12.98	†	ns
Beck's Depression Inventory II	-8.94	19.07	†	-1.02	12.25	ns	†
Positive Negative Affect Schedule - Positive	1.82	14.50	ns	-1.53	18.55	ns	†
Warwick-Edinburgh Mental Well-being Scale	3.90	10.19	**	2.68	10.18	ns	*

Note. Participants are included as having likely PTSD if they scored > 32 on the pre-training baseline measure of the Impact of Event Scale Revised. Scores reflect the change in mental health state after training relative to baseline measures prior to training (post – pre, POMP scores). Significance of Time reflects a paired samples student t-test between pre and post training. Significance of Group is derived from independent samples student t-tests between Suppress-Negative and Suppress-Neutral groups on the post - pre values. The p-value is indicated as such: < 0.001(***), < 0.01(**), < 0.05(*), <= 0.1(†), > 0.1(ns).

Table S10.

A. *For High Trait Anxious Participants Only, the Relative Risk of Worsening Mental Health After Training (Post-Pre or Followup – Pre) in the Suppress-Negative Condition Compared to the Suppress-Neutral Condition on Immediate and 3-Month Assessments (significant reductions in bold).*

	**Immediate**	**Delayed**
State Trait Anxiety Inventory – State	1.22 [0.65, 2.28]	1.11 [0.57, 2.13]
Penn State Worry Questionnaire	1.22 [0.50, 2.97]	1.35 [0.66, 2.76]
Positive Negative Affect Schedule - Negative	0.73 [0.19, 2.74]	0.89 [0.43, 1.83]
Beck's Depression Inventory II	0.43 [0.18, 1.03]	0.56 [0.25, 1.24]
Positive Negative Affect Schedule - Positive	***0.46 [0.21, 0.98]***	0.73 [0.39, 1.35]
Warwick-Edinburgh Mental Well-being Scale	***0.28 [0.09, 0.87]***	*0.50 [0.25, 1.00]*

Note. Relative risks are italicised if statistically significant. If the relative risk = 1, or if the 95% confidence interval includes 1, then there is no significant difference between the groups.

B. *For Participants with Likely PTSD Only, the Relative Risk of Worsening Mental Health After Training (Post-Pre or Followup – Pre) in the Suppress-Negative Condition Compared to the Suppress-Neutral Condition on Immediate and 3-Month Assessments.*

	**Immediate**	**Delayed**
State Trait Anxiety Inventory – State	0.47 [0.12, 1.86]	0.47 [0.12, 1.86]
Penn State Worry Questionnaire	1.23 [0.34, 4.48]	1.31 [0.44, 3.85]
Positive Negative Affect Schedule - Negative	0.65 [0.15, 2.81]	0.33 [0.04, 2.45]
Beck's Depression Inventory II	0.33 [0.09, 1.23]	0.27 [0.04, 1.97]
Positive Negative Affect Schedule - Positive	0.55 [0.23, 1.27]	0.73 [0.29, 1.80]
Warwick-Edinburgh Mental Well-being Scale	0.18 [0.03, 1.25]	0.16 [0.02, 1.11]

Note. Relative risks are italicised if statistically significant. If the relative risk = 1, or if the 95% confidence interval includes 1, then there is no significant difference between the groups.

Table S11.

A. *Training Benefits on our Delayed Test for Participants with High Trait Anxiety*

Mental Health Questionnaires	**Suppress Negative**		Sig. *Time*	**Suppress Neutral**		Sig. *Time*	Sig. *Group*
	µ	SD		µ	SD		
State Trait Anxiety Inventory - State	-7.32	20.23	ns	-0.89	16.27	ns	ns
Penn State Worry Questionnaire	-8.91	23.38	ns	-3.57	18.75	*	ns
Positive Negative Affect Schedule - Negative	-6.63	19.98	ns	-0.80	15.03	ns	ns
Beck's Depression Inventory II	-9.06	16.42	**	-1.85	14.65	ns	†
Positive Negative Affect Schedule - Positive	7.72	20.56	*	-1.79	15.45	ns	†
Warwick-Edinburgh Mental Well-being Scale	5.75	18.42	†	-1.40	14.21	ns	ns

Note. Participants are included as having high anxiety if they scored > 44 on the pre-training baseline measure of the Trait portion of the State Trait Anxiety Inventory. Scores reflect the change in mental health state 3 months after training relative to baseline measures prior to training (follow-up – pre, POMP scores). Significance of Time is derived from paired samples student t-tests between pre-training and follow-up. Significance of Group is derived from independent samples student t-tests between Suppress-Negative and Suppress-Neutral groups. The p-value is indicated as such: < 0.001(***), < 0.01(**), < 0.05(*), <= 0.1(†), > 0.1(ns).

B. *Training Benefits on our Delayed Test for Participants with Likely PTSD*

Mental Health Questionnaires	**Suppress Negative**		Sig. *Time*	**Suppress Neutral**		Sig. *Time*	Sig. *Group*
	µ	SD		µ	SD		
State Trait Anxiety Inventory - State	-13.03	23.47	*	-1.67	15.37	ns	ns
Penn State Worry Questionnaire	-5.91	24.54	ns	-8.89	17.07	†	ns
Positive Negative Affect Schedule - Negative	-9.55	24.18	*	-3.89	15.39	ns	ns
Beck's Depression Inventory II	-17.88	18.56	**	-6.48	13.57	†	†
Positive Negative Affect Schedule - Positive	10.68	19.97	ns	-2.22	16.62	ns	ns
Warwick-Edinburgh Mental Well-being Scale	13.47	19.91	*	-1.29	14.74	ns	†

Note. Participants are included as having likely PTSD if they scored > 32 on the pre-training baseline measure of the Impact of Event Scale Revised. Scores reflect the change in mental health state 3 months after training relative to baseline measures prior to training (follow-up – pre, POMP scores). Significance of Time is derived from paired samples student t-tests between pre-training and follow-up. Significance of Group is derived from independent samples student t-tests between Suppress-Negative and Suppress-Neutral groups. The p-value is indicated as such: < 0.001(***), < 0.01(**), < 0.05(*), <= 0.1(†), > 0.1(ns).

Table S12.

Subjective Reports About the Experience of Suppressing Fearful Thoughts Over 3 Months, as a Function of Post-Traumatic Stress Symptoms. Participants are sorted from highest pandemic-related IES Score to lowest, and divided into quartiles to illustrate that perceptions of usefulness and likelihood of future use were greatest in the most symptomatic participants.

PTSD/Depression/Anxiety: Pre-training scores (POMP) for IES-R, BDI-II, and STAI-Trait respectively.

Usefulness: Do you think that the suppression (mind-blanking) strategy you learned through the course of the training was useful or helpful to use outside the lab in your own life?

Reusability: How likely are you to re-use the suppression (mind-blanking) strategy you learned to decrease your anxiety or thoughts about the negative events in your life?
Rating Scale: Very unlikely - 1; Unlikely - 2; Neutral - 3; Likely - 4; Very likely - 5

Impact: In your own words, please elaborate on your opinion about re-using the suppression (mind-blanking) strategy in the previous question.

ID	PTSD	Depression	Anxiety	Useful?	Reusability?	Impact
234	69.32	53.33	71.67	Yes	3	Need to remember to do it in the moment
158	68.18	28.33	38.33	Yes	3	I like the method but often I find it difficult to remember that I can use it for situations outside of the ones I wrote down
80	67.05	33.33	66.67	Yes	5	The mind-blocking is able to prevent someone from concentrating on negative events and just focus on positive and neutral events
128	55.68	35.00	71.67	Yes	5	it’s very useful when trying to get to sleep, because I can easily stop over-thinking
18	51.14	25.00	30.00	Yes	4	Sometimes it just feels impossible to stop thinking if something is worrying you, or you are taken by surprise by a bad event. But if something just begins to creep in you can actively decide to stop.
191	51.14	61.67	76.67	Yes	4	it can definitely help me in having more control over my anxious responses that randomly appear throughout the day
178	50.00	73.33	93.33	Yes	3	help to relax myself for a little while, but anxiety grows later again
61	50.00	45.00	66.67	Yes	5	In practise I couldn't believe how effective it was and it made me realise how powerful my brain can be. I always thought that just dismissing thoughts would make things worse, like 'brushing it under the carpet' however, it’s a useful tool as it can put some distressing irrelevant thoughts to bed.

216	47.73	36.67	60.00	Yes	4	I think it has been useful when I have used it in diverting my attention and changing my mood (increase)
106	44.32	5.00	18.33	Yes	3	If a negative effect is strong, the think-blocking is working with other strong thinking so I put my focus on the other thought. The mind-blanking is working just with small negative situations
36	43.18	15.00	48.33	Yes	4	Using the blank mind technique helps to quell the initial panic of a negative thought.
10	35.23	30.00	56.67	Yes	4	It's more useful to do for unlikely events but for events that could happen it is more useful to deal with them head on rather than ignoring them
202	35.23	10.00	33.33	Yes	5	It is a really powerful tool to block worrying thoughts, I will definitely intend to use it again.
209	35.23	15.00	20.00	Yes	4	I think it is useful to do when I have no control over the event and it numbs out the anxiety (anxiety is still there, just less intense)
192	34.09	11.67	21.67	Yes	4	-

First Quarter Summary
PTSD: 49.17 *Depression*: 31.89 *Anxiety*: 51.56 *Usefulness*: 100% *Reusability*: 4.00

197	32.95	10.00	21.67	Yes	4	I think it would be worth trying again in stressful situations. I'm not sure how good I'd be at it though, I think I've forgotten quite a bit since training!
221	32.95	13.33	25.00	Yes	4	I think it was helpful in approaching my stresses and anxieties about potential events in a different way and did help me manage the way I thought about hypothetical/future events
226	32.95	18.33	33.33	Yes	4	It feels to me like I did not "practice" enough yet as my daily job was quite intense, full of meetings and with very little personal time to thinks about pleasant or unpleasant things. I don't always block fears because they are usually related to something that I can act on. I "push back" only things I cannot do anything about i.e. death of a friend
215	31.82	10.00	36.67	Yes	3	I think it is a good strategy but it is so easy to forget about it when you’re in a low mood
218	30.68	23.33	35.00	Yes	5	It is a great way to prevent bad thoughts and make me feel better, as well as not wasting time worrying about something before it even happens.
8	29.55	6.67	10.00	Yes	4	It's worth trying if I'm having negative thoughts but it doesn't work if I'm feeling really sad or worried about something
134	28.41	28.33	20.00	Yes	5	Since having the training, I have used the strategy a lot and found it to be very effective in stopping me thinking about negative or disturbing ideas or situations
180	28.41	5.00	11.67	Yes	4	Not sure if it is quite the same, but I am quite good at compartmentalizing things and manage to separate home and work (so can blank out things when I am not in work or home mode)

3	27.27	21.67	45.00	Yes	4	Mind blanking - helps to focus on the day-to-day activities rather than worrying on events.
7	26.14	11.67	25.00	Yes	4	I think the method of using mind-blanking is a very effective 'circuit breaker' to limit the likelihood of myself or an individual spiralling into negative thoughts. Thus, I wouldn't be able to say as to whether I consciously use it however, I hope and feel that the method encourages me to pause and think more rationally when I am experiencing negative or worrying thoughts.
239	26.14	6.67	38.33	Yes	4	I think it might be a good strategy to use, if one can remember to do it in the "heat of the moment"
145	26.14	16.67	66.67	No	2	I have never applied it before, so I just don't do it now. I usually deal with my fears by talking about them.
15	25.00	56.67	73.33	Yes	4	I use it to stop me dwelling on it at the time although thoughts do sometimes come back but is useful in the present moment
176	25.00	53.33	38.33	Yes	4	I think it could be useful, since I have a tendency to let negative thoughts take over my mind. Using this technique, I try to stop my negative thoughts before they spiral out of control.
135	22.73	23.33	43.33	Yes	3	a good technique, but doesn't feel natural

Second Quarter Summary

PTSD: 28.41 *Depression*: 20.33 *Anxiety*: 34.89 *Usefulness*: 93% *Reusability*: 3.87

170	22.73	26.67	55.00	Yes	5	There are certain events that I worry about, triggered by situations. I have frequently, in those situations, used the suppression strategy to good effect. Eg. overtaking cars on the highway is always stressful, but visualizing a car crash is not exactly helpful in that moment, and this helps to keep my mind clear and on the road. Suppression doesn't work well when I'm worrying about something like getting a paper published (or not), because I feel like the worry should help me to work towards my goal, not be suppressed.
225	22.73	11.67	28.33	Yes	4	It is a way to do not worry about future events we do not have any control. The example than members of my family are going to die for age will happens and thinking about that is not going to change the sadly fact, another negative effects I can affect the outcome and blocking them it is not useful because we can act about them and change the outcome
210	21.59	15.00	13.33	Yes	4	The strategy could be accompanied by self-talk aimed at telling oneself not to worry unnecessarily.
26	21.59	18.33	48.33	Yes	3	I tried using the strategy a few times soon after the initial study but have not used it much since
88	21.59	15.00	48.33	Yes	5	I think that it was a good strategy to learn and try to apply, but outside the lab I found it hard to make myself really do that and remind myself of the strategy

140	21.59	18.33	28.33	Yes	4	It worked well to stop negative thoughts becoming intrusive
168	20.45	40.00	51.67	Yes	4	I know there are things which could very well happen, but thinking about them doesn't do me any good, e.g. I know worrying about deadlines doesn't make me do more work. So, it's better for me to just force myself to not think about it.
173	20.45	8.33	45.00	Yes	4	For temporary cases it works to suppress immediate emotions but if the source of anxiety is still there it will come back until the source is dealt with. It's not a long-term fix to getting rid of anxiety but it may help you feel not feel anxious as much of the time.
233	20.45	21.67	35.00	Yes	3	I think it's important to be aware of the problem and understand and accept how you feel about it, because I don't believe that suppress the emotions is good to solve the problems. If you understand and accept how you feel first then yes you want to stop your mind from starting a loop of negative emotions that won't lead you anywhere
149	19.32	15.00	23.33	No	3	I think I don't have a ton of negative thoughts in general, and so I sort of forgot to use the strategy. There are a few specific negative thoughts that I have that are recurrent, and it might be a useful technique to deal with these
190	19.32	31.67	63.33	Yes	4	I think this technique is useful for things out of your control, but may not be the best approach for worries that require solutions. Some problems need to be confronted in order to be overcome
154	18.18	10.00	25.00	Yes	5	By using suppression, negative thoughts can be prevented from being brought to the conscious mind so it is quite useful for future negative events which I am not even sure would happen or not.
46	18.18	25.00	25.00	Yes	4	When negative thoughts enter my mind, I am more likely to actively try to suppress or 'blank' them
147	15.91	41.67	56.67	Yes	4	I was able to do this and I could tell the anxiety decreased so I will use - when I remember to and it is remembering to that is the issue... I need to connect the anxiety triggers with the resource of blanking and putting that in place.
203	15.91	10.00	20.00	No	3	Potentially it could help but I have forgotten to try it! I will try to use it more and see.

Third Quarter Summary
PTSD: 20.00 *Depression*: 20.56 *Anxiety*: 37.78 *Usefulness*: 87% *Reusability*: 3.93

174	14.77	3.33	30.00	Yes	4	good strategy. another tool in my tool belt.
141	13.64	6.67	15.00	Yes	4	I think it's a great way to learn to control bad thoughts you have about life and to help improve mood.
111	12.50	11.67	18.33	Yes	4	I think it is a useful strategy and it has been good to have this reminder as I will now use it more

29	11.36	6.67	35.00	Yes	4	I think it is a useful task to control negative thoughts and stop them escalating.
153	11.36	10.00	45.00	Yes	3	-
235	10.23	10.00	33.33	Yes	3	It was useful tool to stop bad thoughts from coming to mind
56	10.23	8.33	11.67	Yes	5	I think I could use it to help with food cravings or negative self-image thoughts rather than using it to block anxiety about events occurring.
2	9.09	6.67	20.00	No	3	I hadn't really thought about it over the past 3 months. I don't tend to dwell on too many negative events that much, and when I do I tend to think through them quite a lot rather than blanking them out. If in the future my worries are becoming more problematic (constant), then I might use the suppression strategy.
94	9.09	5.00	15.00	Yes	4	It is a useful skill in not over-thinking issues, particularly during a pandemic when lots of things are out of our control.
11	7.95	3.33	23.33	No	1	I don't think suppression of thought is a good thing as I feel you need to be honest with your feelings even if they are detrimental to your mental health.
126	6.82	35.00	51.67	Yes	4	I caught myself sometimes utilizing the mind-blanking when irrational worries arose. This gave me a short-lived alleviation of the anxiety.
144	3.41	1.67	3.33	Yes	4	Helpful, but more of a supplementary tool for me as I already dealt with anxiety well.
162	1.14	15.00	25.00	Yes	3	It's a very interesting process, but it is not automatic to put it in place and I feel that thinking a little about negative events is sometimes important, at least to think of solutions and alternatives. I think it is a very good solution for recurrent, nagging negative thoughts.
16	0.00	8.33	33.33	No	3	did not use it
17	0.00	5.00	13.33	No	4	Useful for worries for things for which I have no control over such as worrying about things that haven't happened yet (e.g. deaths in the family) or things that might not happen (e.g. another lockdown), however potentially not productive to completely mind-blank on the issue because there could still be actions that I could take - such as spend more time with my grandparents, try to look for another job/take provisions for possible lockdown etc. So, I think I could use it to some extent but not completely mind-blank on issue/subject of worry.
167	0.00	46.67	73.33	No	3	I find it wouldn't be very helpful for me since I prefer to think through problems rather than blank them out.

Fourth Quarter Summary

PTSD: 7.60 *Depression*: 11.46 *Anxiety*: 27.92 *Usefulness*: 69% *Reusability*: 3.50
